# Efficient multi-robot exploration using deterministic and metaheuristic methods

**DOI:** 10.3389/frobt.2026.1781310

**Published:** 2026-09-28

**Authors:** Faiza Gul, Imran Mir, Manzar Abbas, Agostino Forestiero

**Affiliations:** 1 Department of Electrical Engineering, Air University, Aerospace and Aviation, Kamra Kalan, Pakistan; 2 Department of Avionics, Air University, Aerospace and Aviation, Kamra Kalan, Pakistan; 3 Institute for High-Performance Computing and Networking, National Research Council of Italy, University of Calabria, Rende, Italy

**Keywords:** crayfish optimizer, exploration, multi-robots, optimization, perception and autonomy, stochastic methods

## Abstract

Autonomous exploration is a critical challenge in the use of robots across many applications. Nonetheless, current methodologies exhibit inadequate exploration rates, principally attributable to limited global coverage and infrequent decision-making. To mitigate these restrictions, we offer EMREL (Efficient Multi-Robot Exploration), a hybrid optimization method intended to enable rapid multi-robot exploration in intricate, unfamiliar environments. Our methodology implements the deterministic Coordinated Multi-Robot Exploration (CME) formulation, with cost and utility values organized into a Frontier Information Structure (FIS) shared across all robots. The deterministic cost and utility initially direct the prioritization of neighboring cells surrounding each robot. Subsequently, the stochastic optimizer (Crayfish Optimizer) enhances search space by improving overall solutions. This suggests that robots first evaluate their environment using a deterministic method before moving forward under the control of a stochastic algorithm. We evaluated the suggested hybrid technique on both ordinary and complicated maps and compared it with contemporary algorithms. The simulations show that the proposed method outperforms the contemporary algorithms in exploration rate, temporal efficiency, and number of failed simulation runs. These research results demonstrate the potential of EMREL as a versatile, superior, and efficient approach for robotic exploration, establishing a framework for potential developments in multi-robot system design.

## 1 Introduction

In the study of robotics, one of the most challenging tasks is the exploration of unknown environments. From disaster relief missions to extraterrestrial exploration, the ability to navigate and map unfamiliar terrain is paramount for success. While single robots have demonstrated remarkable capabilities in controlled environments, the complexity and uncertainty inherent in uncharted territories necessitate the use of multi-robot systems. In response, multi-robot exploration (MRE) systems have developed as a viable alternative, facilitating cooperative exploration tactics that enhance task efficiency and resilience in dynamic situations. Multi-robot systems employ distributed intelligence, enabling individual robots to collectively map unfamiliar terrains, share exploratory duties, and improve task completion rates. These systems enable concurrent environmental coverage, hence decreasing mission duration and improving resistance to system faults.

In multi-robot systems, inter-robot cooperation is a pivotal element affecting total performance. Efficient collaboration necessitates real-time data transmission to avert collision conflicts and reduce unnecessary area scans (Amigoni et al., 2017; Queralta et al., 2020). This coordination is accomplished using shared mapping frameworks and adaptive path-planning algorithms, allowing each robot to optimize its trajectory based on dynamic environmental data. These techniques provide accelerated exploration rates, more precision in environmental reconstruction, and augmented fault tolerance in situations including communication disruptions or hardware failures. Notwithstanding the benefits of MRE, enhancing multi-robot cooperation in uncertain and dynamic environments continues to be a challenging issue. The discipline has progressively concentrated on formulating optimization-driven strategies, especially swarm intelligence techniques, to improve exploration efficacy and task distribution. The efficacy of MRE systems directly influences practical applications, such as environmental monitoring, planetary navigation, and automated reconnaissance. Thus, enhancements in robotic exploration algorithms directly result in more scalable and deployable robotic solutions. The primary challenge in MRE is devising exploration strategies that optimize terrain coverage while reducing redundant traversal and wasteful path selection, rendering it a complex multi-objective optimization problem.

In the last few years, the field of multi-robot exploration has achieved significant advancements. Scholars have investigated a range of methodologies, such as machine learning-based approaches, swarm intelligence techniques, and decentralized control algorithms. These methods effectively use the combined intelligence of robot teams to negotiate challenging terrain. Furthermore, developments in sensor technologies such as inertial measurement units, LiDAR, and cameras have improved robot vision and enabled them to collect extensive environmental data in real time. When combined with developments in mapping and localization algorithms, these sensors allow robots to create comprehensive, self-navigating maps of unfamiliar areas.

As of now, there are many proposed algorithms for mobile robot exploration. These algorithms can be classified as deterministic or stochastic. Deterministic algorithms have proven to be reliable methods. These algorithms are usually unable to provide reliable results in situations where there is incomplete information, unpredictable terrain characteristics, sensor noise, communication delay, and non-linear environmental actions. Deterministic methods operate within known and predefined operating conditions and thus do not have the flexibility and robustness necessary to operate in real time in extraterrestrial environments where there is little or no prior knowledge present. The drawbacks of deterministic algorithms are poor adaptability, brittle performance, and failure to produce strong behaviors under uncertainty. These issues are critical in missions that demand real-time flexibility and toughness in undertaking activities like search and rescue, environmental monitoring, inspection of hazardous sites, and underground exploration.

Metaheuristic algorithms are methods that use a random concept. The conventional algorithms have relatively low accuracy and convergence rates, which makes them susceptible to falling into the local optima problem easily. Moreover, these algorithms involve the generation of non-unique solutions and therefore require benchmark cases, which unfortunately most optimization problems do not have. Reluctantly, randomness in the results is encountered, causing suboptimal solutions. Although the existing meta-heuristic algorithms are available, they have been utilized for single robot applications, and their impact on multi-robot configuration is yet to be ascertained. Furthermore, these algorithms have limited scalability and adaptability in dynamic and obstacle-cluttered environments.

Considering the exploration approaches, the frontier based (Yamauchi, 1997) and the coordinated multi-robot explorations (CME) (Burgard et al., 2005) are predominantly most viable solutions for one robot and a group of robots, respectively. CME, a frontier-based framework that coordinates several robots using a cost-and-utility evaluation of candidate frontier cells. The utility of a cell decreases when claimed by other robots, encouraging the team to spread across the environment rather than converge on the same target. In this work, our hybrid approach is built around the cost-and-utility formulation of CME as the deterministic core.

The proposed method incorporates two mutually supportive elements. The deterministic element is based on Coordinated Multi-Robot Exploration (CME), whose cost and utility formulas are structured into a shared Frontier Information Structure (FIS) that maintains occupancy probabilities, utility values, and frontier flags across the robot team. The stochastic element, controlled by the Crayfish Optimizer, predicts each robot’s subsequent motions by functioning on the candidate cells exposed through the FIS, enabling robust exploration of unknown environments.

Therefore, we present **EMREL**, a hierarchical framework for **E**fficient **M**ulti-**R**obot **E**xp**L**oration in complex situations. As exploration involves three major challenges: i) no prior map, ii) partial or no observability, and iii) multi-robot coordination under uncertainty. As conventional information gain methods often treat the frontier points (boundary points) independently, it can cause redundant exploration, inefficient exploration rates, and multiple robots selecting nearby regions. We use CME because it aids robots in understanding global exploration structure; it also prevents robots from choosing spatially correlated regions of targets, and the decisions are made on structure information, not only on raw utility score, and it also avoids random fluctuations in exploration rate. In the next step, the stochastic optimizer drives each robot’s local decisions, allowing the team to escape local optima where deterministic methods alone fail.

We compared our strategy to both classical and cutting-edge simulation approaches. The results show that our strategy consistently completes exploration in a reduced time and with enhanced accuracy in all circumstances. Refer to Figure 1 for an overview of the proposed framework. Here is a summary of the work’s findings:

1. CME’s shared cost and utility values are incrementally modified with each new sensor observation, enabling real-time decisions by every robot.
2. A hybrid approach combining CME with the crayfish optimization algorithm is proposed to enhance coverage and evade local optima in unknown environments.
3. The method is evaluated through MATLAB simulations and ROS-based implementation using multiple test scenarios.

**FIGURE 1:**
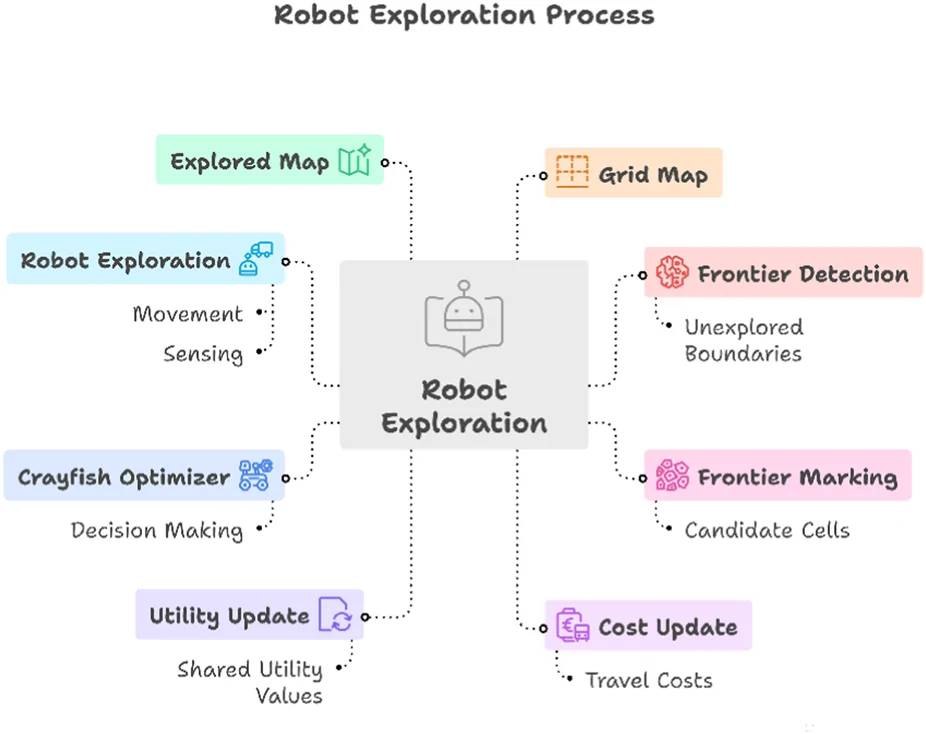
An introduction of the potential exploration framework.

Our research primarily supports random optimization because of its flexibility in multi-robot exploration. In dynamic and often discontinuous situations, establishing a smooth, differentiable cost function is difficult, hindering the efficacy of gradient-based approaches. Random optimization excels in such contexts by efficiently handling non-continuous search spaces and avoiding local optima. It enables robots to equilibrate exploration and exploitation, facilitating efficient path selection while preserving adaptability in uncertain environments.

### 1.1 Motivation

Hybridized algorithms are the newest trend with a variety of uses and benefits, according to the literature review and cited publications (Gudivaka et al., 2024; Romeh et al., 2023; Kamalova et al., 2019). Competitive hybrid algorithms that generate the best results with the least amount of computation are created by combining several algorithms. The development of new hybrid algorithms has all been directed towards solving the limitations identified in prior algorithms and improving the optimization variables while exploring various complex environments, as evidenced by the listed articles. Therefore, it is imperative to create innovative hybrid strategies that address spatial constraints and integrate bio-inspired approaches with the CME framework. We ensured that various workspace sections are examined at a relatively equal pace in our suggested approach.

### 1.2 Research contribution

The implementation of the recently created crayfish algorithm, which is especially set up for multi-robot space exploration in an unknown environment, is presented in this work. In our approach we make sure that different workspace locations are checked at roughly the same speed so that some locations are not checked much later than others. For efficient communication between agents, an intelligent initial configuration is incorporated. The outcome shows that the suggested method efficiently and quickly investigates the unfamiliar environment. Lastly, the simulation has shown how well the suggested crayfish optimizer performs. The efficiency of the method is examined in several workspace regions. The obtained results are examined and contrasted with other cutting-edge techniques that have been documented in the literature.

### 1.3 Paper organization

The remainder of the sections in this paper are organized as follows: Section 2 reviews relevant literature, highlighting both opportunities and challenges associated with multi-robot exploration in unknown space. In Section 3, the detailed explanation of deterministic methods is discussed along with mathematical formulas. Section 4 presents the main procedure of the Crayfish Optimizer, which is designed to solve the space exploration problem along with the formation of the proposed approach. In Section 5, detailed experiments and results are discussed. Section 6 provides the performance of the proposed EMREL with contemporary algorithms and final analysis and discussion. Section 7 discusses the integration of the proposed method with ROS2. The conclusion and future work are presented in Section 8.

## 2 Related work

Recent developments in MRE have gained considerable focus because of various applications across multiple domains. These changes have been especially important in human-robot collaborative systems, where autonomous robots help people explore new areas that are too dangerous or difficult for them to reach on their own. With such robotic systems, different tasks can be performed with greater efficiency and efficacy, such as rescue missions, large-scale environmental monitoring, etc. Multi-robot systems, which are usually controlled by humans, strengthen these qualities substantially by encompassing larger areas more effectively and requiring little help from outside. Because of this, research in this field has concentrated more and more on finding the best ways to deploy and coordinate these robotic systems so that they can work more efficiently and be more flexible. The fundamental advantage of multi-robot systems over single-robot systems involves potential coordinated exploration in any challenging environment. Compared to a single robot that should independently explore and visualize its environment, a multi-robot system can distribute the assignment among multiple agents, which enables simultaneous information acquisition and increased area coverage. This distributed strategy substantially decreases mission duration and improves the robustness of exploration tasks. Nevertheless, guaranteeing efficient cooperation among multiple robots poses several challenges, specifically in the exchange of information, collision avoidance, and inefficient scanning.

In order to handle multi-robot systems, two different fundamental control strategies have been examined: centralized and decentralized control. Centralized techniques utilize a single control unit, which supervises all robots in the system, permitting accurate communication but resulting in flexibility constraints and susceptibility to a single point of failure. On the contrary, decentralized control (especially in swarm robotics) permits autonomy at the standalone robot level, which in turn contributes to improved versatility and fault tolerance. Swarm robotic systems, influenced by natural social behaviors, rely on interpersonal connections between robots and their surroundings, which renders them extremely versatile to dynamic conditions. Despite developing swarm behavior offering added complexities, utilizing collaborative abilities and distributed computation decreases computational demands on individual robots, boosting general system efficiency. In Table 1, the latest major studies are summarized based on the numerical techniques and metaheuristic algorithms in the mobile-robot exploration.

**TABLE 1:** The nature-inspired approaches in the mobile-robot exploration.

Description	Environment type	Optimization	Exploration	Source
Adjusted velocity and coarse direction parameters for the optimal solution	Indoor, structured maps	Numerical Optimization	Social potential field, multi-robot system	Fang et al. (2004)
Information-driven POMDP framework that jointly controls multi-robot formation geometry and drives robots toward actions that maximally reduce uncertainty about victim locations in SAR missions	Disaster/rescue environments: partially observable, uncertain, dynamic indoor spaces	An investigation of MRS coordination about mobility and multi-robot task planning	MCTS/point-based POMDP solvers, Formation geometry optimization, Joint reward: info gain + formation cost	Chang et al. (2026)
Hierarchical multi-robot exploration framework using RegionGraph, a weighted graph of unexplored spatial regions, enabling global-to-local task decomposition and asynchronous robot operation, achieving a 20% efficiency gain	indoor, structured maps	Weighted graph construction, Greedy/heuristic local allocation	Region Graph region-based exploration	Meng et al. (2025)
Advanced Multi-Objective Salp Swarm Algorithm Exploration Technique (AMET), which is a novel optimization framework designed to enhance the efficiency and robustness of multi-robot exploration	Indoor, structured maps	Numerical and metaheuristic optimization	Frontier method, SSA	El Romeh and Mirjalili (2025)
Combines Coordinated Multi-Robot Exploration (CME) and African Vultures Optimization Algorithm (AVOA) to optimize the construction of a finite map in multi-robot exploration	Indoor, structured maps	Numerical and metaheuristic optimization	Frontier method, AVOA	Romeh et al. (2023)
Exploration of Unknown Space	Indoor, structured maps	Numerical and metaheuristic optimization	Frontier method, SSA	Romeh and Mirjalili (2023)

Regarding the optimization of robotic exploration, techniques can be essentially categorized into deterministic and stochastic approaches. Deterministic methods depend on predetermined rules and established decision-making standards, guaranteeing that the identical input will perpetually generate the same outcome. Although such techniques provide reliability and computational effectiveness, they frequently encounter problems with versatility in high-dimensional or rapidly evolving environments, where they can end up stuck in local optima. On the contrary, stochastic algorithms, specifically metaheuristic methods, embed unpredictability into their search process, which enables increased exploration variety and a better opportunity to avoid local optima. Metaheuristic techniques, influenced by physiological and social processes, offer more reliable outcomes for large-scale and intricate environments but at the expense of increased computational expenses and fluctuating convergence rates. Despite these limitations, their innate capability to swiftly adapt exploration strategies renders them more appropriate and well-suited for multi-robot systems functioning in unexplored and dynamic environments. Preliminary investigations in multi-robot exploration validated the fundamental concepts of task allocation, coordination, and territorial coverage, establishing the foundation for contemporary optimization techniques. As examination developed, the combination of heuristic and meta-heuristic techniques substantially improved the versatility and efficiency of MRE methodologies, which strengthened their significance to actual-world issues such as environmental surveillance, manufacturing automation, and autonomous reconnaissance. The aforementioned developments demonstrate the expanding significance of versatile, modular, and innovative algorithms in addressing the constraints of conventional rule-based methods.

## 3 Deterministic methods

In autonomous robotics research, lately there has been a substantial transition toward multi-robot exploration. As conditions become more complex, effective collaboration between numerous autonomous vehicles is vital to ensure effective exploration. Deterministic techniques have frequently played a significant role in addressing this particular problem by offering rule-driven assessment techniques that maximize the distribution of exploration tasks. These methods attempt to equilibrate the trade-off involving computational productivity and exploration efficiency, thereby guaranteeing that robots methodically navigate unknown environments while reducing ineffective motions.

A frontier information structure is frequently employed in robotic exploration when a robot must investigate an unfamiliar region. As the robot navigates, it constructs a map of the environment utilizing techniques such as simultaneous localization and mapping. In this map, a frontier denotes the demarcation between regions that the robot has previously explored and regions that remain uncharted. The frontier regions are significant, as advancing towards them enables the robot to get novel knowledge about the surroundings. The frontier information structure enables the robot to catalog and arrange frontier locations, assessing which should be explored next based on criteria such as distance and prospective information yield. This methodology enables robots to navigate areas effectively and is extensively utilized in autonomous exploration systems.

Robots frequently depend on occupancy grid maps to precisely depict explored and unexplored regions, dividing the environment into distinct cells that indicate the probability of being free, occupied, or unknown. This representation provides the essential structure required for finding and evaluating frontier areas during exploration.

### 3.1 Coordinated multi-robot exploration

The mobile robot team utilizes multi-robot exploration as the searching method, where the team starts from entire uncertainties to a finite map. The data obtained can be used with different algorithms for exploration. Initially, the robot is placed in an indoor environment without any knowledge about its surroundings. The robot is equipped with a sensor with a limited coverage range. The sensor discovers frontier cells for finite map creation in unknown space. The environment is represented by an occupancy grid map under the CME. The occupancy grid map contains numbers reflecting the probability of an obstacle in each grid cell as well as the utility and travel cost of each cell. The occupancy grid map is based on only a few cells around the robot due to the restricted coverage of the sensor; an illustration is shown in Figure 2. The cost-minimizing exploration calculates the distance needed for each robot to reach the frontier point.

**FIGURE 2:**
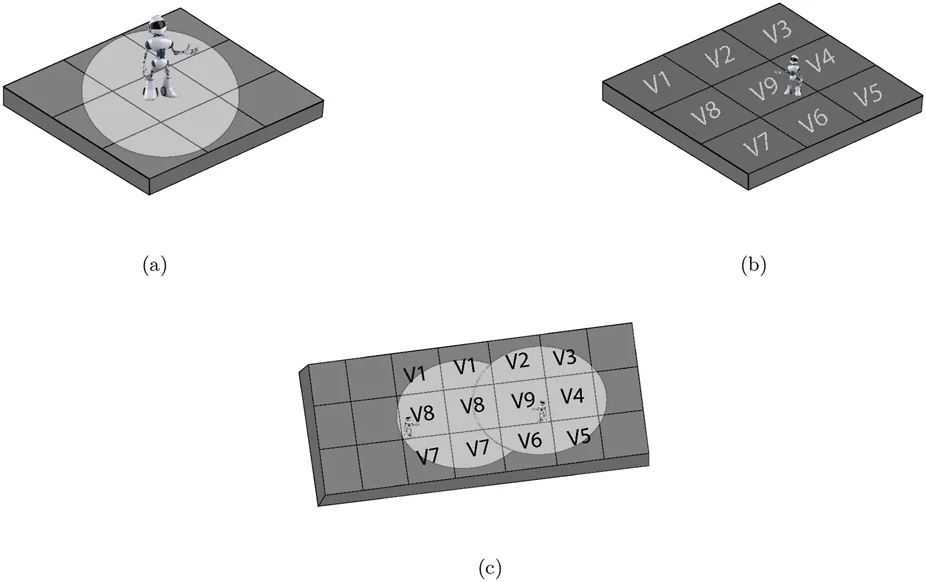
Graphic representation of grid cells utilizing sensor aspect. **(a)** Eight vector cells viewed through a sensor. **(b)** Robot positioning on nine vector. **(c)** Cells Robot motion across vector cells.

### 3.2 Occupancy grid map

Elfes invented the Occupancy Grid Map (Elfes, 1989), which is a popular data structure for representing the environment in robotics. This map is essentially a 2D matrix, in which each cell represents a distinct area of the environment. Figures 2A–C, 3 depict a sample grid map. In the occupancy grid representation, a cell c is categorized into one of three types of states based on its occupancy probability P (occ_c): free (P close to 0), occupied (P close to 1), or unknown (P = 0.5). A frontier cell is formally defined as any free cell that is adjacent to at least one unknown cell. Formally:

$$\begin{array}{l} F=\left\{\left.c\in G\;\right|\;P\left({occ}_{c}\right)\approx 0,\;\exists \;c^{\prime }\in N\left(c\right)\text{ such that }\right.\left.P\left({occ}_{c^{\prime }}\right)=0.5\right\} \end{array}$$

**FIGURE 3:**
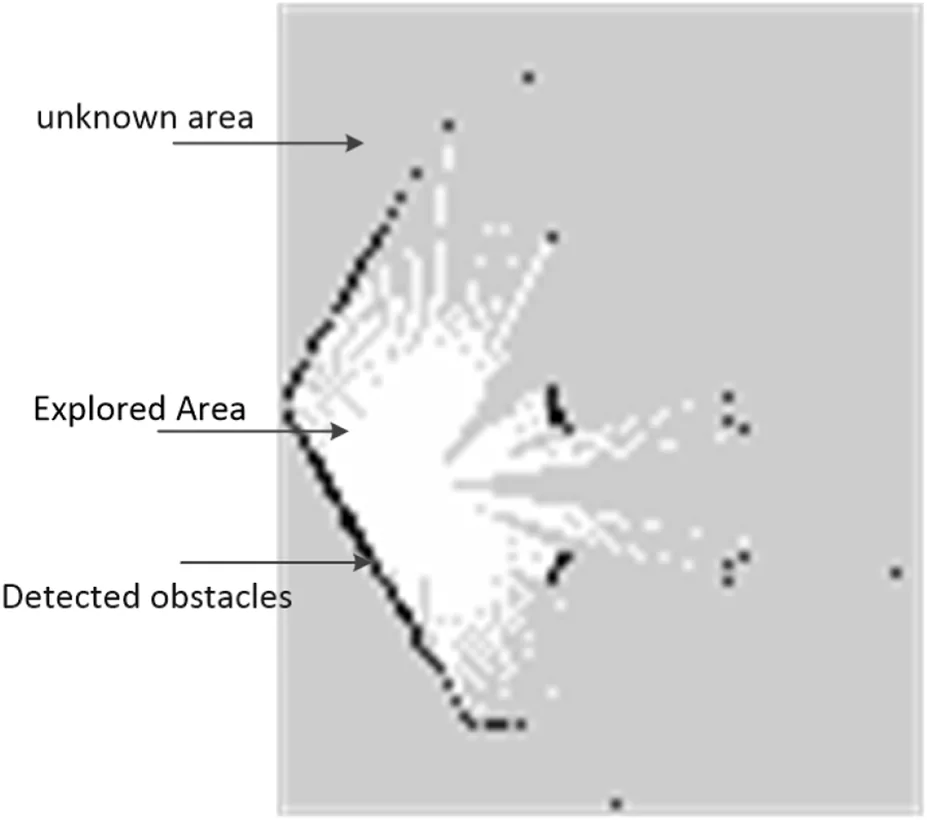
An illustration of occupancy grid maps. White pixels denote the investigated region; grey pixels signify the undiscovered region; black pixels indicate the detected barriers.

where G denotes the occupancy grid, N(c) denotes the eight-neighborhood of cell c, and F denotes the set of frontier cells at any given iteration.

As a result, the cells in a grid can be labeled as

$$C_{i,j}=\left\{\begin{array}{cc} Occupied\;if\;Occ\left(C_{i,j}\right)>0 & \\ Unknwon\;if\;Occ\left(C_{i,j}\right)<0 & \\ UnOccupied\;if\;Occ\left(C_{i,j}\right)=0 & \end{array}\right.$$ (1)

$Occ\left(C_{ij}\right)$ denotes the occupancy log odds from Equation 1. We select 0 as the threshold value since logodds (0.5) = 0.

### 3.3 Formulation of cost function

We frequently frame the exploration of unexplored areas as an optimization issue, with the goal of allocating an occupancy probability to each cell within the map. To accomplish this, the robot must optimize the examined area while reducing energy expenditure. The robot’s sensor, utilizing an occupancy grid map, encompasses many surrounding cells. The exploration cost for each cell is calculated based on criteria including occupancy probability, Euclidean distance, and sensor observation (Equation 2). We add the previous cost of a previously explored cell to the current cost of the new position. However, if the cell is a frontier cell and the sensor’s ray beams have just discovered it, there is no backward cost from earlier stages (Equation 3). The occupancy probability value $\left(P\text{\_}occ_{xy}\right)$ for an unidentified cell is 0.500. Cells occupied by obstacles possess occupancy values nearing 1, whereas metrics approaching 0 signify a high degree of certainty that the cell is free of obstacles. According to Equation 2 in the original work (Burgard et al., 2005), the occupancy values decrease as the sensors reach farther-away cells.

$$\begin{array}{l} V_{x,y}=min\left\{V_{\left(x+\Delta x,y+▵y\right)}+\sqrt{\Delta x^{2}+\Delta y^{2}}.P\left(occ_{x+\Delta x,y+\Delta y}\right)\right\} \end{array}$$ (2)

$$V_{x,y}=min\left\{\sqrt{\Delta x^{2}+\Delta y^{2}}.\;P\left(occ_{x+\Delta x,y+\Delta y}\right)\right\}$$ (3)

where, Δx,Δy ∈ [−1,0,1]  and $P\left(occ_{x+\Delta x,y+\Delta y}\right)\in \left[0,occ_{\max}\right]$ . The objective of cost minimization is to ascertain the cell with the lowest cost among adjacent cells, signifying the ideal subsequent position for a robot. In a single-robot system, identifying the least cost may suffice to ascertain the robot’s subsequent action. In a multirobot system, coordination and collaborative organization are essential for effective exploration.

### 3.4 Utility value

The utility value measures the exploration status of each cell in the grid map. Initially, all cells are allocated uniform utility values, which are then modified throughout the exploration process. As robots navigate their surroundings and reach frontier regions—the demarcations between explored and unexplored territories—the value of these frontier cells decreases. This decline enables robots to focus on unexplored areas by steering their movement towards cells with increased utility values, thus reducing unnecessary revisiting of cells. The expense linked to each grid cell is dictated by its proximity to the robot’s present location. Additionally, the utility of a border cell is influenced by two factors: (1) the local environmental characteristics in its neighborhood, and (2) the quantity of robots simultaneously approaching it. By adjusting their trajectories to target cells for optimum benefit, robots autonomously facilitate the exploration of unexplored territories. This approach provides new environmental data while progressively enhancing the common map coverage, promoting efficient and coordinated exploration. The mathematical formalization of the interaction between utility-driven decision-making and adaptive path planning is presented in Equation 4, which captures the equilibrium between exploration incentives and collaborative resource distribution.

$$U_{j}^{i}=U_{j-1}^{i}-\Sigma P\left(\left\|occ_{x,y}^{u}-occ_{x,y}^{u^{\prime }}\right\|\right)$$ (4)

The utility value of a cell is represented by $U_{j-1}^{i}$ , which represents its previous exploration phase, and $U_{j}^{i}$ , which represents its current condition. The robot’s position and exploring progress affect the utility value of each cell. The robot’s current position is deducted from the cell’s occupancy probability to update the utility value. This modification ensures that cells with greater occupancy probabilities have lower usefulness, whereas unexplored or less populated cells guide the robot to new locations. Using Equation 5, the robot selects the cell with the highest exploration efficiency for its next move.

$$\left(j,i\right)=max\left(U_{j}^{i}-V_{x,y}\right)$$ (5)

## 4 Crayfish optimization algorithm: COA

The foraging, rivalry, and summertime vacationing habits of crayfish serve as an inspiration for COA (Jia et al., 2023). The summer resort stage reflects COA’s exploring phase, whereas the stages of foraging and competition indicate its exploitation phase. The COA method starts with creating the crayfish colony X in its first phase to mimic the characteristics of swarm intelligence optimization.

### 4.1 Initialization phase

The COA algorithm’s initialization procedure entails the generation of a collection of potential solutions, represented by **X**, refer to Equation 6.

$$X=lb+\left(ub-lb\right)\times rand,$$ (6)

In this case, it rand produces a random number between 0 and 1. ub is the upper bound and lb is the lower bound. In every cycle, each X produces a candidate solution. The optimal solution achieved is regarded as the minimal solution.

The behavior of crayfish alters in reaction to temperature variations. The temperature is determined using Equation 7. In the COA, crayfish demonstrate heat avoidance behavior when temperatures surpass 30 °C. In contrast, they exhibit foraging behavior at temperatures below 30 °C. Crayfish have pronounced foraging behavior at temperatures ranging from 20 °C to 30 °C, with an ideal feeding temperature of 25 °C. Consequently, the COA delineates the temperature range as 20 °C–35 °C. The equation controlling crayfish eating behavior is denoted as Equation 8.

temp=rand×15+20 (7)

$$P=C_{1}\times \left(\frac{1}{\sqrt{\left(2\pi \right)}}\times \exp\left(-\frac{{\left(temp-25\right)}^{2}}{2\mu^{2}}\right)\right)$$ (8)

μ and $C_{1}$ are two controlling factors to control the intake of crayfish at different temperatures.

### 4.2 Heat avoidance behavior

In the heat avoidance behavior phase, crayfish recognize temperatures above 30 °C as excessively high. In reaction, they search for a comparatively cooler environment to evade the heat. The mathematical model that describes crayfish summer behavior is expressed as an equation; refer to Equation 9.

$$X_{\mathrm{cave}}=\left(X_{\mathrm{Lbest}}+X_{\mathrm{Gbest}}\right)/2$$ (9)

where; $X_{\mathrm{Gbest}}$ is the ideal position of the current population, and $X_{\mathrm{Lbest}}$ is the best position of the crayfish as determined by the number of iterations.

Two scenarios arise throughout the summer vacation period. In the first situation, when the random value (rand) is below 0.5, signifying the lack of other crayfish in the cave, the crayfish will promptly enter the cave to evade the heat. Equation 10 specifies the mathematical model that underlies this behavior. In the second case, when rand is greater than or equal to 0.5, crayfish exhibit competitive behavior towards one another. Equation 11 represents this competitive behavior quantitatively.

$$X_{\mathrm{new}}=X_{i,j}+\left(2-\frac{t}{T}\right)\times rand\times \left(X_{\mathrm{cave}}-X_{i,j}\right)$$ (10)

$$X_{\mathrm{new}}=X_{i,j}-X_{z,j}+X_{\mathrm{cave}}$$ (11)

where *t* is the current iteration number and *T* is the maximum iteration number. $X_{z,j}$ The agent is randomly selected from the crayfish.

### 4.3 Foraging behavior

Crayfish are able to forage when the temperature is 30 °C or lower. In this situation, the crayfish will advance towards the food source. Upon locating the meal, the crayfish will evaluate its size using Equation 12. When the food is considered excessively huge, the crayfish will utilize a particular eating strategy: it will fragment the food into smaller portions with its claws, thereafter consuming it in an alternating manner with its second and third claws.

$$Q=C_{2}\times rand\times \frac{f_{X}}{f_{\mathrm{Xbest}}}$$ (12)

The equation delineates its variables as follows:

$C_{2}$ signifies the food factor, which is the predominant food with a constant value of 3; $f_{X}$ symbolizes the fitness value of the crayfish and $f_{\mathrm{Xbest}}$ represents the fitness value of the optimal global location.

When the requirement $Q>\left(C_{2}+1\right)/2$ is satisfied, the crayfish demonstrates a particular foraging behavior. In this circumstance, the crayfish dismembers the meal into smaller fragments and subsequently ingests it in an alternate manner utilizing its second and third claws. Equation 13 that regulates this behavior is articulated as follows:

$$\begin{array}{ll} & X_{i,j}=X_{i,j}+\exp\left(-\frac{1}{Q}\right)\times P\times X_{\mathrm{Gbest}}\\ & \;\times \left(sin\left(2\times \pi \times rand\right)-cos\left(2\times \pi \times rand\right)\right) \end{array}$$ (13)

When Q≤(C2+1)/2 , the crayfish merely needs to advance towards the food and consume it immediately, as represented by Equation 14:

$$X_{\mathrm{new}}=\left(X_{i,j}-X_{\mathrm{Gbest}}\right)\times P+P\times rand\times X_{i,j}$$ (14)

### 4.4 Intelligent initial configuration (IIC)

Robotics is a field where Intelligent Initial Configuration (IIC) is very important, especially for jobs that need optimization and decision-making. In robotics, we strategically implement initial configuration, initial settings, or starting behaviors to enable robots to commence their tasks quickly and successfully. Robotic systems can automatically set up themselves in a way that makes it easier to navigate and complete tasks. They can do this by learning from past successful paths or by using algorithms that simulate different initial configurations.

In the same way, IIC is crucial in robotic swarm systems, where many robots work together to achieve a single goal. This controls where, what, and how each robot starts and communicates. The swarm can work better and more efficiently by intelligently assigning initial configurations based on factors like how close they are to the job, their range of skills, and their ability to communicate. This allows the swarm to coordinate its operations from the beginning.

First, let’s examine a single-objective optimization problem (SOP) in which finding an optimal solution $\left(x^{*}\right)$ that maximizes or minimizes a designated objective function f(x) is the goal. In this case, SS represents the search space of the SOP, and each solution is identified as $x_{i}$ within X , where $i=1,2,\ldots ,N_{i}$ denotes the total number of solutions. Every possible solution, $x_{i}$ , stands for a potential contender to address the SOP.

IIC is a collection of previously determined optimal or nearly optimal solutions obtained from earlier algorithm iterations. The algorithm is constantly updating this database as it finds new potential optimal solutions. The database is represented $DB_{t}$ during the th iteration of the algorithm, and it consists of a collection of solutions $s_{1},s_{2},\ldots ,s_{n}$ , where each $s_{i}$ represents a highly performing solution found in a previous iteration.

The IIC module inside the EMREL algorithm creates the initial population for the algorithm’s th iteration by using the solutions kept in $DB_{t}$ . In this procedure, solutions $DB_{t}$ are chosen at random and used as a basis for creating new solutions by small perturbations. By introducing diversity using a mutation operator, this method makes it easier to explore different areas of the search space.

To generate a population (refer to Figure 4), a few steps are followed, which are

- We choose *k* solutions at random, where *k* is less than *N* from $DB_{t}$ , and we refer to this chosen collection of solutions as $S_{t}$ .
- Apply a mutation operator to each solution $s_{i}$ in *St* to create a new solution $s_{i0}$ for each $s_{i}$ .
- The initial population $P_{t}$ for the *th* iteration of the algorithm is made up of all new solutions $s_{10},s_{20},\ldots ,s_{k0}$ .

**FIGURE 4:**
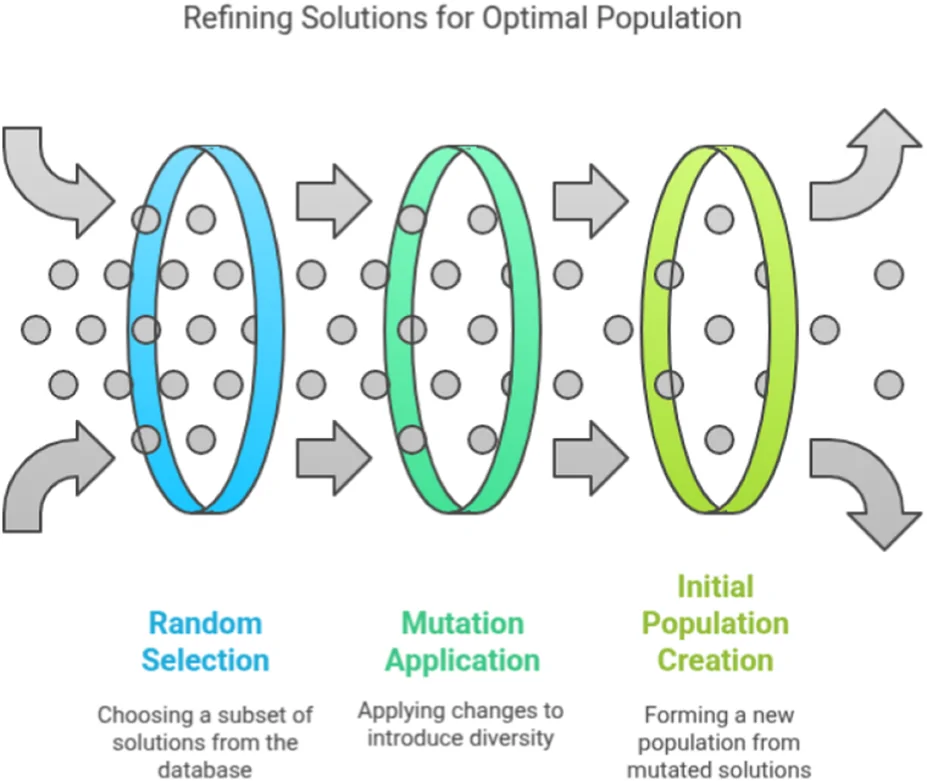
Visualization of refining solutions.

This method uses the information present in past successful solutions to direct the search during its first stages. The mutation operator adds diversity to the initial population, preventing it from simply repeating previously solved problems. This variety encourages investigation throughout the search field. So, the IIC part improves the quality of the solutions that the EMREL algorithm comes up with while also making convergence go much faster.

### 4.5 Formation of EMREL

The EMREL framework implements the deterministic method (CME) together with the stochastic method (COA).

The COA transfers biological activities like “summer vacation” (exploration) and “foraging” (exploitation) into mathematical phases to increase multi-robot exploration. The temperature-based model governs transitions between these stages, balancing global search and local refinement. Model parameters, such as movement thresholds and competition dynamics, are set based on empirical investigation. The method initiates with the establishment of grid maps, subsequently identifying the next optimal moves through a probabilistic framework.

The grid map cells are assigned a utility value of 1. The sensor’s range limits the exploration, which results in eight potential cells surrounding the robot that the sensor monitors (refer to Figures 2b,c) forming the **“crayfish”** (**Cf**). The crayfish, designated as Cf1 through Cf8, serve as candidates for the robot’s next move; refer to Figure 5 for the robot decision-making process. The robot then allocates a cost to each candidate cell and subtracts the utilities from the costs of the adjacent eight grid cells using a defined formula.

$$\begin{array}{ll} & CfPos_{i,j}^{t+1}=P_{\left(occ_{i+\Delta i,j+\Delta j}\right)}^{i,j}+\left(2-\frac{t}{T}\right)\\ & \;\times rand\times \left(X_{\mathrm{cave}}-P_{\left(occ_{i+\Delta i,j+\Delta j}\right)}^{i,j}\right) \end{array}$$ (15)

$$\begin{array}{ll} & CfPos_{i,j}^{t+1}=P_{\left(occ_{i+\Delta i,j+\Delta j}\right)}^{i,j}+\exp\left(-\frac{1}{Q}\right)\times P\times X_{\mathrm{Gbest}}\\ & \;\times \left(sin\left(2\times \pi \times rand\right)-cos\left(2\times \pi \times rand\right)\right) \end{array}$$ (16)

**FIGURE 5:**
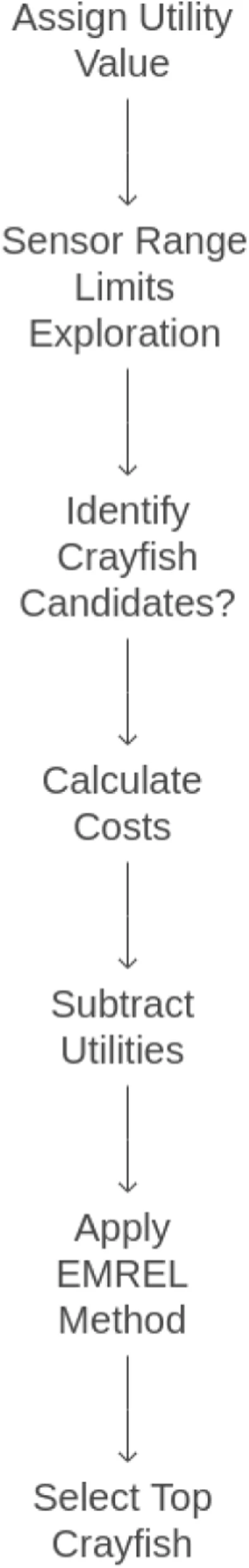
Robot exploration and decision-making process.

Upon calculating the new positions through the Equations 15, 16, the EMREL method determines the three crayfish exhibiting the highest utility values. We establish the prioritization of these elements based on their revised positions and Q values. Figure 6 illustrates the workflow of the crayfish exploration technique with CME and intelligent initial configuration. The exploration process then transitions into an iterative loop, identifying the target cell coordinates for each robot, calculating cell costs, and systematically updating utilities. All necessary iterations conclude the exploration process. The EMREL method shows a structured and useful way to explore with multiple robots, effectively dealing with the difficulties that come with starting points and other natural limitations.

**FIGURE 6:**
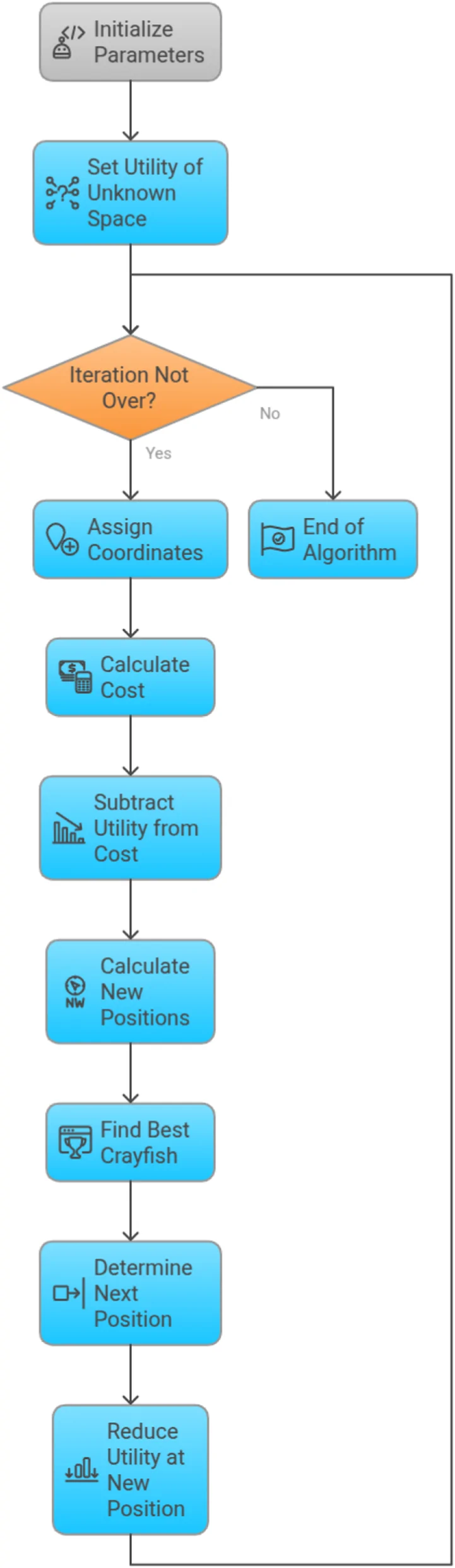
EMREL algorithm flowchart.

## 5 Results and discussion

We evaluated the effectiveness of our technique using a two-dimensional simulated environment. Figure 7a depicts the exact test conditions used in our investigations. The method for cooperative autonomous exploration in a multi-robot context completes the task successfully, even when some robots fall into local/global minima/maxima. The environment spans 900 by 900 grids and incorporates a range of barriers, each with unique features that accurately reflect real-world conditions. In this configuration, black represents obstacles, while white represents passable spaces.

**FIGURE 7:**
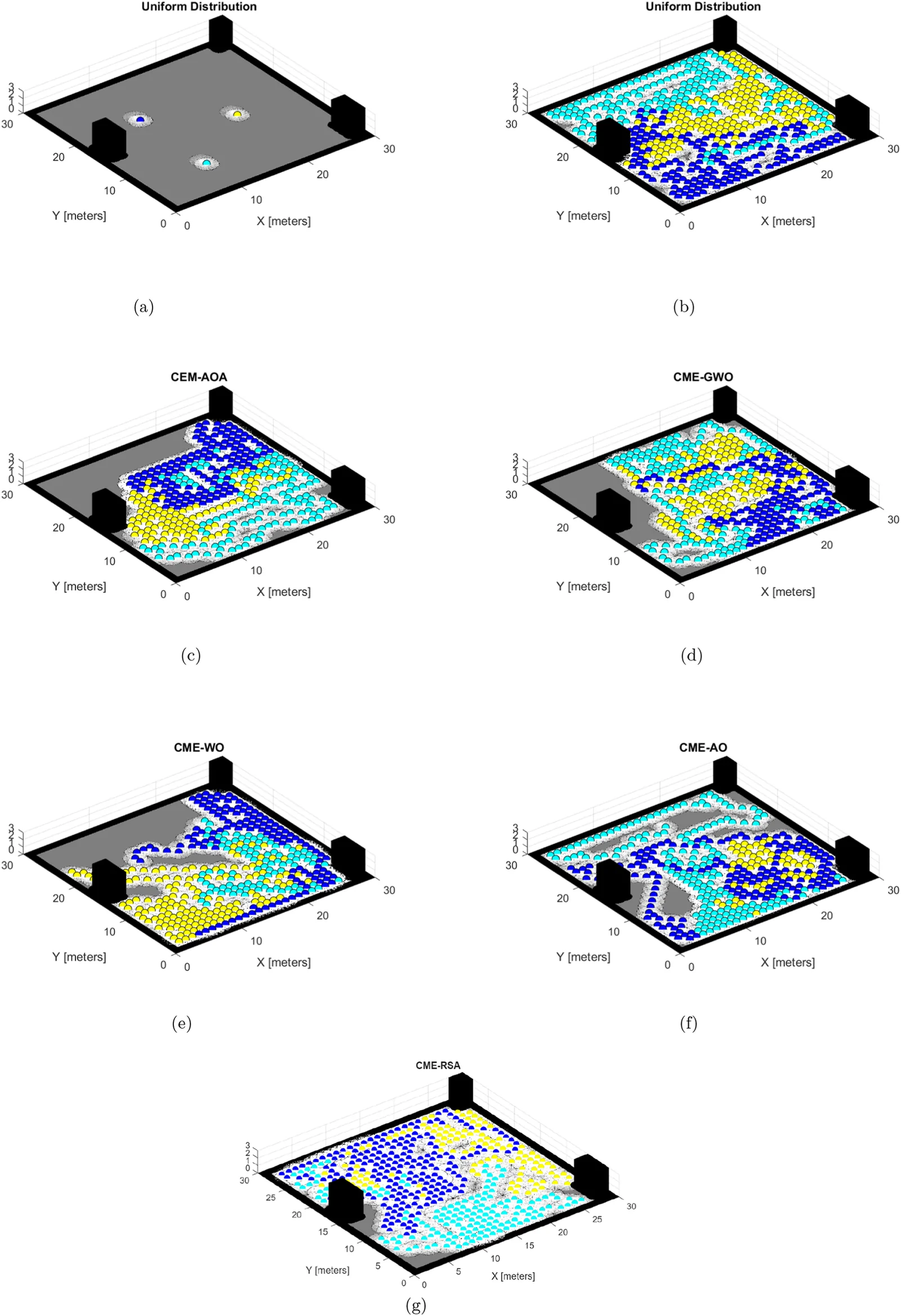
Uniform distribution of obstacles for EMREL and contemporary algorithm. **(a)** Uniform distribution. **(b)** EMREL 99%. **(c)** CME-AOA 74%. **(d)** CME-GWO 89%. **(e)** CME-WO 82%. **(f)** CME-AO 87%. **(g)** CME-RSA 96%.

It is tough to assess the effectiveness of stochastic algorithms in multi-robot exploration due to their intrinsic complexity and stochasticity. To precisely evaluate the efficacy of techniques put into practice, extensive testing in a variety of settings, from simple to complex, is necessary.

Our research empirically analyzes the EMREL with Intelligent Initial Configuration (IIC). This method combines a unique technique for figuring out the initial configuration of robots. The EMREL method optimizes exploration efficiency across several initial start configurations, outperforming conventional methods. Uniform distribution, strategic positions, random placements, and clustered position locations are some of these configurations.

We compare the effectiveness of EMREL with five other hybrid approaches: coordinated multi-robot exploration with Arithmetic Optimizer (CME-AOA), coordinated multi-robot exploration with Grey Wolf Optimizer (CME-GWO), coordinated multi-robot exploration with Whale Optimizer (CME-WO), coordinated multi-robot exploration with Aquila Optimizer (CME-AO), and coordinated multi-robot exploration with Reptile Search Optimizer (CME-RSA). This comparison analysis focuses on three major performance parameters: the number of unsuccessful runs, the percentage of the examined region, and the execution time.

To quantify the explorative performance of each method, we utilized a measure denoted as *‘M’*, which represents the exploration rate. This rate is defined as follows:

$$M=\frac{U_{t}-U_{e}}{U_{t}}$$

where ‘M’ signifies the utility of the explored area and ‘T’ stands for the overall utility of the uncharted area. This metric efficiently assesses each algorithm’s effectiveness based on the level of exploration attained.

The studies were conducted using uniform settings throughout all simulations on a map with a consistent dimension of 30 m × 30 m, and the sensor ray length is 1.5 m. We displayed the map in a grid style, using blue, cyan, and yellow colors to represent the three robots, a gray area to represent the unexplored area before the exploration process, and black cells to represent the obstacles.

For our research, we created four separate cases, each specifically designed to evaluate a different aspect of the algorithms’ functionality.

Case 1: In this basic case, all approaches were assessed from a standard starting point on an unaltered map arrangement. Its goal was to provide a basic performance standard for every technique.

Case 2: This scenario adds dynamic obstacles that move over time, but the map structure stays unchanged. This evaluates the algorithms’ capacity to instantly adjust and reorganize their plans in response to shifting environmental circumstances.

Case 3: Building on the first case, the map’s arrangement stayed the same, but EMREL’s starting points were changed. The purpose of this scenario was to evaluate how well EMREL performed under various initial conditions and how flexible it was.

Case 4: In this case, the beginning positions were continuously adjusted, but the map layouts stayed constant. We conducted this to assess the techniques’ adaptability to various terrain features and issues. In addition, we ran tests with different starting positions in more complex scenarios to compare our method to current algorithms, demonstrating its effectiveness.

### 5.1 Case 1: uniform distribution

In the first case of our investigation, we set a baseline to compare the performance of three exploration methods: Efficient Multi-Robot Exploration (EMREL), Coordinated Multi-Robot Exploration with Arithmetic Optimizer Algorithm (CME-AOA), Coordinated Multi-Robot Exploration with Grey Wolf Optimizer (CME-GWO), Coordinated Multi-Robot Exploration with Whale Optimizer (CME-WO), Coordinated Multi-Robot Exploration with Aquila Optimizer (CME-AO), and Coordinated Multi-Robot Exploration with Reptile Search Optimizer (CME-RSA). We test each approach under identical conditions, which include a uniform starting position and 250 iterations on a static map measuring 30 m × 30 m. We uniformly deploy three robots around the map, starting in various sections to reduce overlap and increase coverage. We intend this configuration to ensure fair comparisons between the methods. Figure 7 depicts the results of this scenario visually. Figure 7a depicts the initial robot distribution, whereas Figure 7b represents the uniform distribution for the EMREL method. Figures 7c–f contain the explored region percentage for contemporary algorithms.

### 5.2 Case 2: dynamic obstacles

Figures 8, 9 show the visual representation of the simulation results for the dynamic case for the EMREL algorithm. It consists of six subfigures with labels 8a through 8d and 9a to 9d. Every subfigure represents a different aspect of the case: it depicts the robots’ dynamic movement dispersal and the advancement in exploration that a specific approach achieves. In these simulations, our strategy outperformed other alternatives in terms of exploration efficiency and space coverage. This demonstrates the method’s high versatility and efficacy in dynamic and complicated contexts.

**FIGURE 8:**
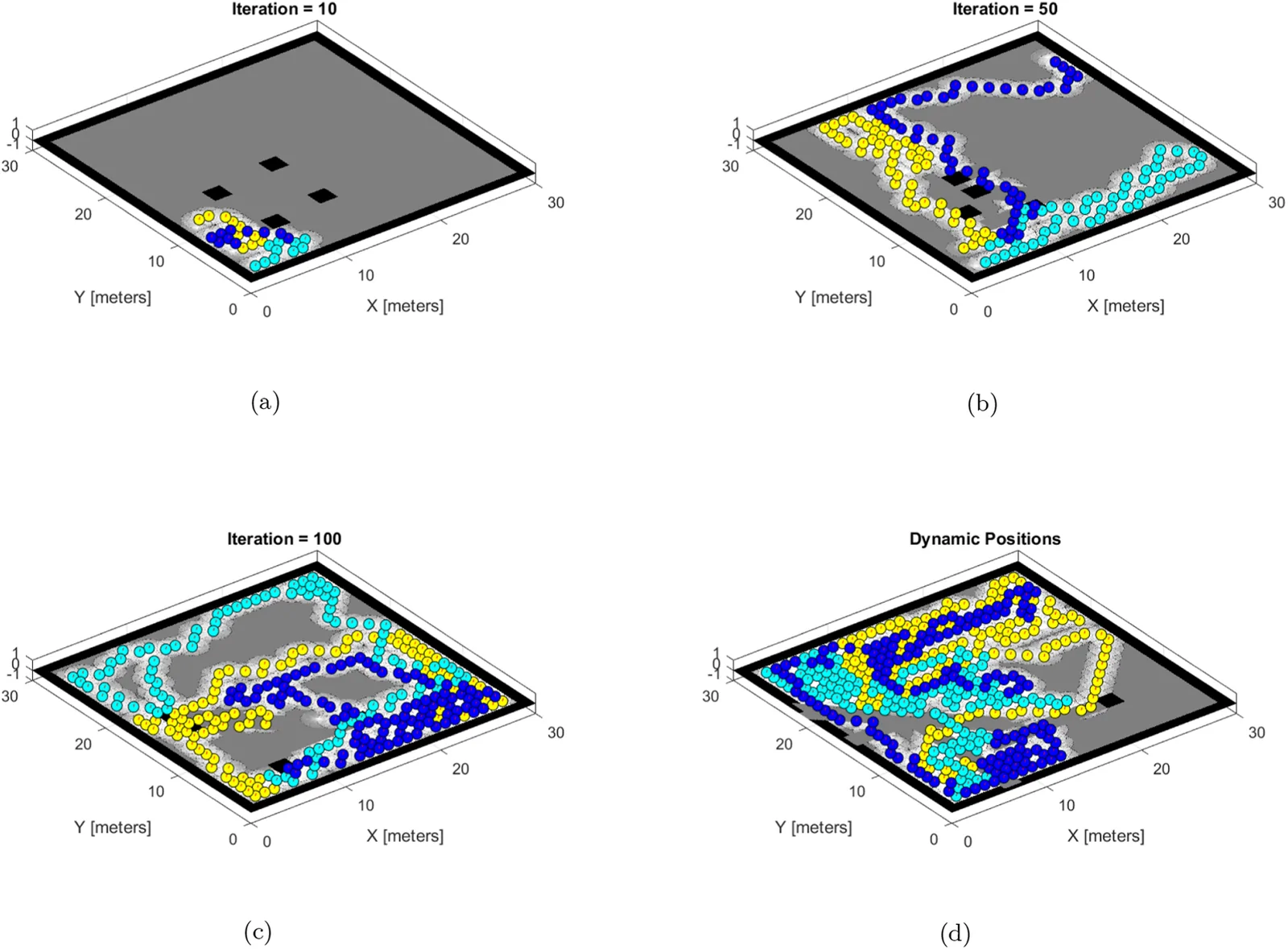
Map1: dynamic movement of obstacles for EMREL algorithm. **(a)** Iteration # 10. **(b)** Iteration # 50. **(c)** Iteration # 100. **(d)** Iteration # 250.

**FIGURE 9:**
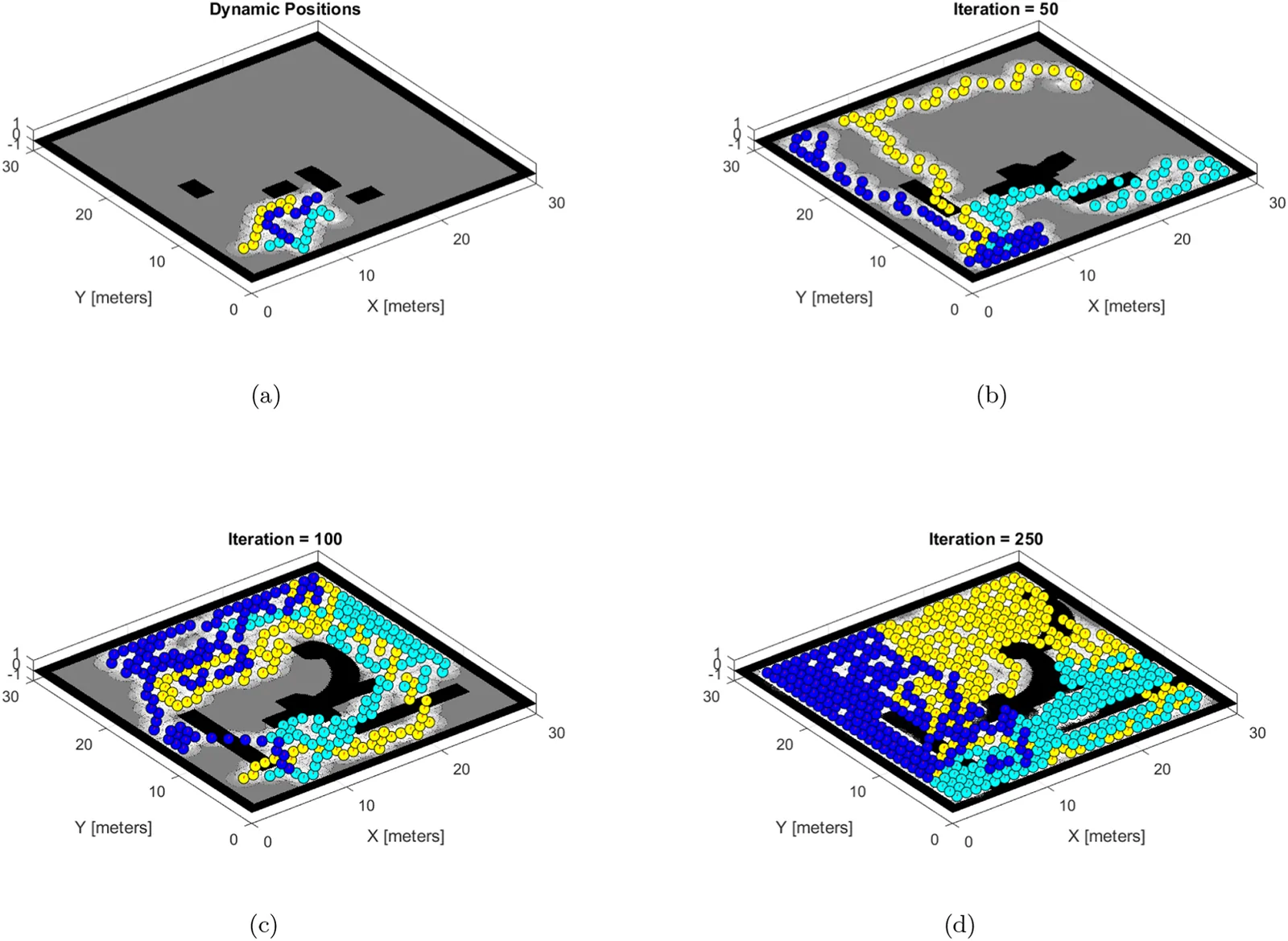
Map2: dynamic movement of obstacles for EMREL algorithm. **(a)** Iteration # 10. **(b)** Iteration # 50. **(c)** Iteration # 100. **(d)** Iteration # 250.

### 5.3 Case 3: adaptability to different starting positions

The third case adds another level of intricacy to the assessment procedure. The map structure remains unchanged despite the addition of different beginning places. In this test setting, we can see how long-lasting and flexible the suggested method is when starting with smart configurations, as well as how changes in starting configurations affect the efficiency of exploration.

We investigated three different starting positions: strategic, random, and clustered places. Ten subfigures overall, representing the exploratory processes related to each of these combinations, were displayed in Figures 10–12. Each pair of these subfigures gives a snapshot of the robots’ initial positioning and subsequent EMREL exploration performance under that particular configuration.

**FIGURE 10:**
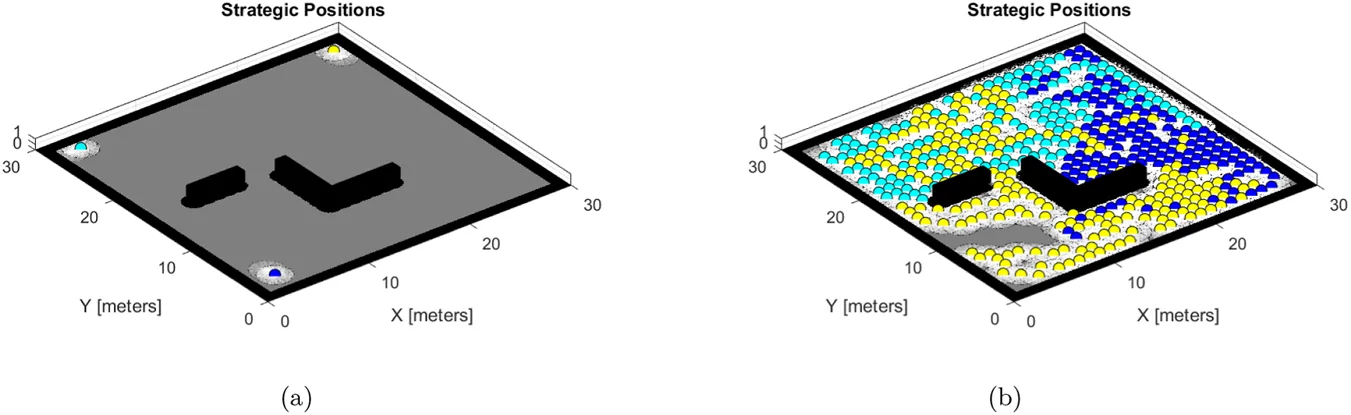
Strategic positions of obstacles for EMREL algorithm. **(a)** Strategic positions of robots. **(b)** Strategic positions EMREL 98.54%.

**FIGURE 11:**
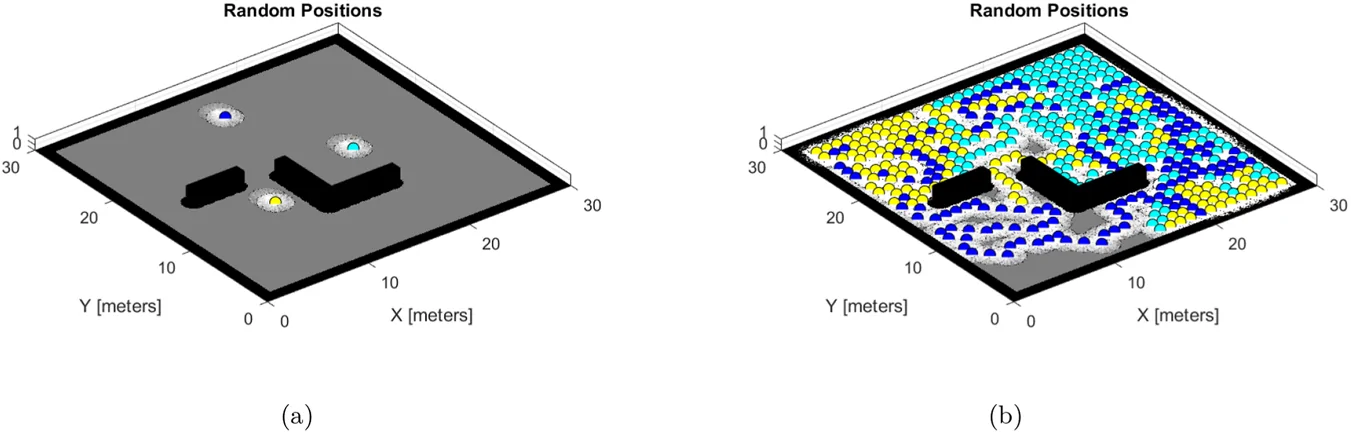
Random positions of obstacles for EMREL algorithm. **(a)** Random positions of robots. **(b)** Random positions EMREL 93.72%.

**FIGURE 12:**
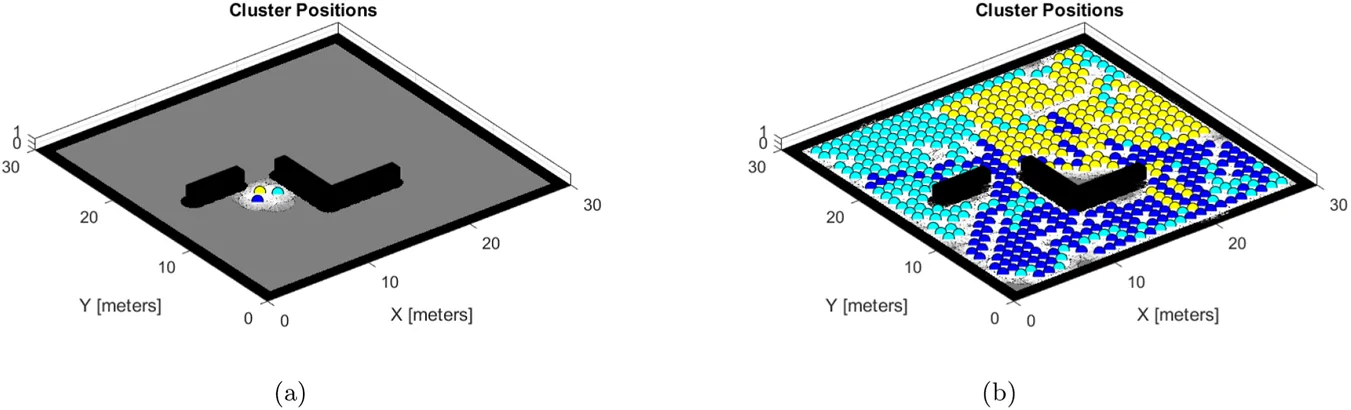
Clustered positions of obstacles for EMREL algorithm. **(a)** Clustered positions of robots. **(b)** Clustered positions EMREL 99.19%.

Strategic Positions (Figures 10a,b): Robots are positioned methodically in key areas, which are carefully selected to guarantee wide coverage and reduce exploration time. The initial configuration of robots during exploration is shown in Figure 10a. Figure 10b displays the impressive exploration efficiency that EMREL attained in this configuration, with an excellent 98.54% exploration rate—the highest of all configurations evaluated in this case study—demonstrating the efficacy of this methodology.

Random Positions (Figures 11a,b): A major problem is navigating through the inherent unpredictability of this setup, where robots are dispersed randomly around the area (Figure 11a). Nonetheless, Figure 11b shows that EMREL was adept at overcoming this barrier. It shows a superior 91.83% exploration rate.

Clustered Positions (Figures 12a,b): The robots were grouped into a single cluster in this design (Figure 12a), which could lead to overlapping exploration tracks and decreased efficiency. However, the EMREL algorithm worked its way through this configuration, achieving an excellent exploration rate of

### 5.4 Case 4: complex map layouts

This case broadens the scope of testing for our multi-robot exploration techniques, focusing on their capacity to adapt to complicated map layouts. The map was characterized by a complex network of passageways and obstacles, necessitating increased strategic planning and cooperation among robots. This case tested the exploration algorithms by simultaneously altering the robots’ starting positions and the environment’s configuration. The method employs incrementally updated frontier information to decrease the duration required for real-time information updates. This enables robots to make exploratory judgments more frequently without depending heavily on quick updates. The stochastic planning approach allows robots to adapt their courses dynamically in response to changes in both global and local environments. We created this simulation to evaluate the algorithms’ flexibility and adaptability in variable and unpredictable settings. Figure 13 depicts the impact of environmental complexity on exploration approaches, highlighting the exploration efficiency of each method in each case of strategic, random, and cluster: Refer to Figures 13–15 for the EMREL, CME-AOA, CME-GWO, CME-WO, CME-AO, and CME-RSA algorithms.

**FIGURE 13:**
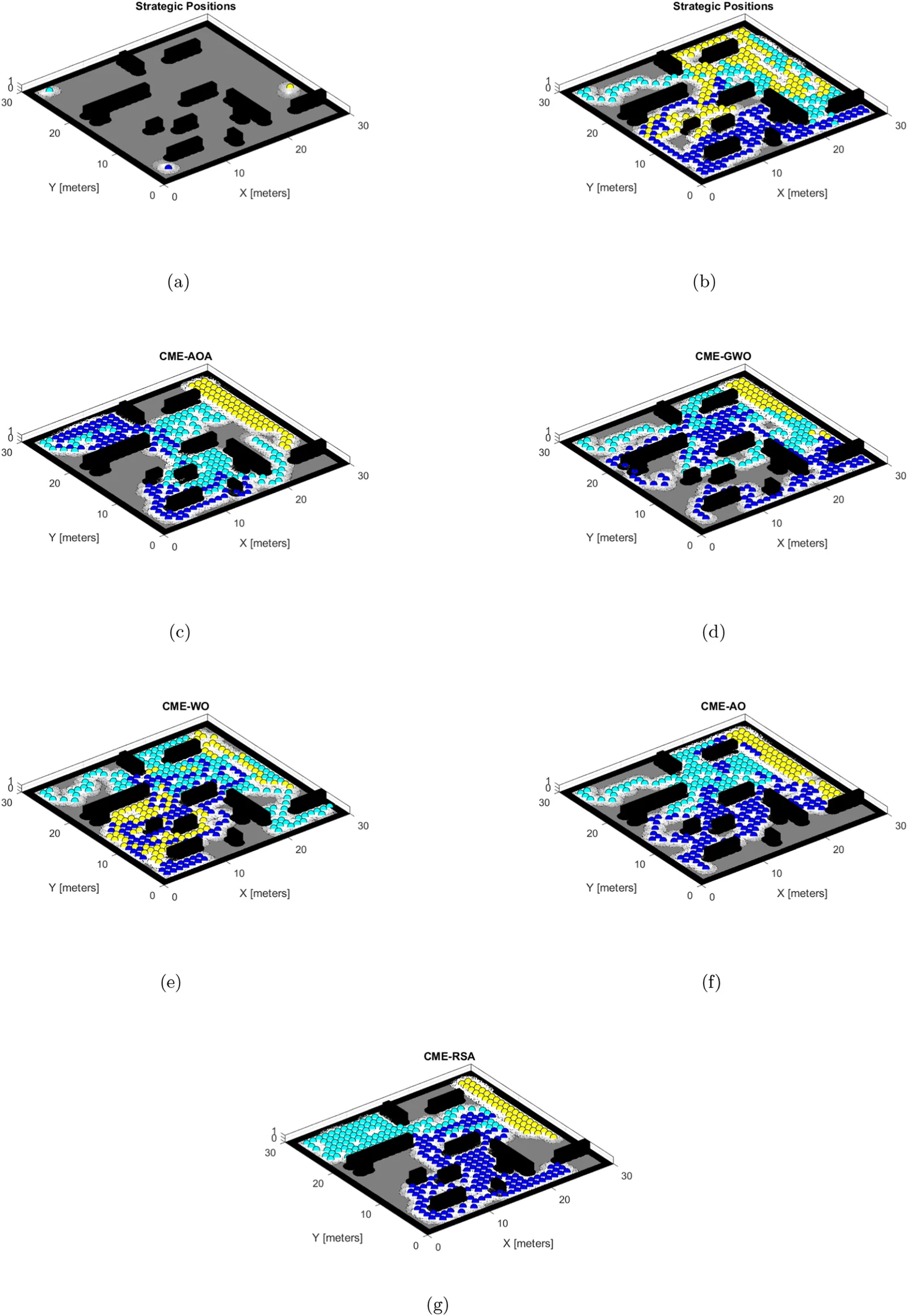
The study focuses on the strategic positions of obstacles in a complex map for both the EMREL Algorithm and the contemporary algorithm. Each color represents a robot in a homogeneous beginning setup, with distinct starting places and travel trajectories in the environment. **(a)**. **(b)** Strategic Positions EMREL 92.59%. **(c)** CME-AOA 72.34%. **(d)** CME-GWO 63.73%. **(e)** CME-WO 85.69%. **(f)** CME-AO 75.45%. **(g)** CME-RSA 65.98%.

**FIGURE 14:**
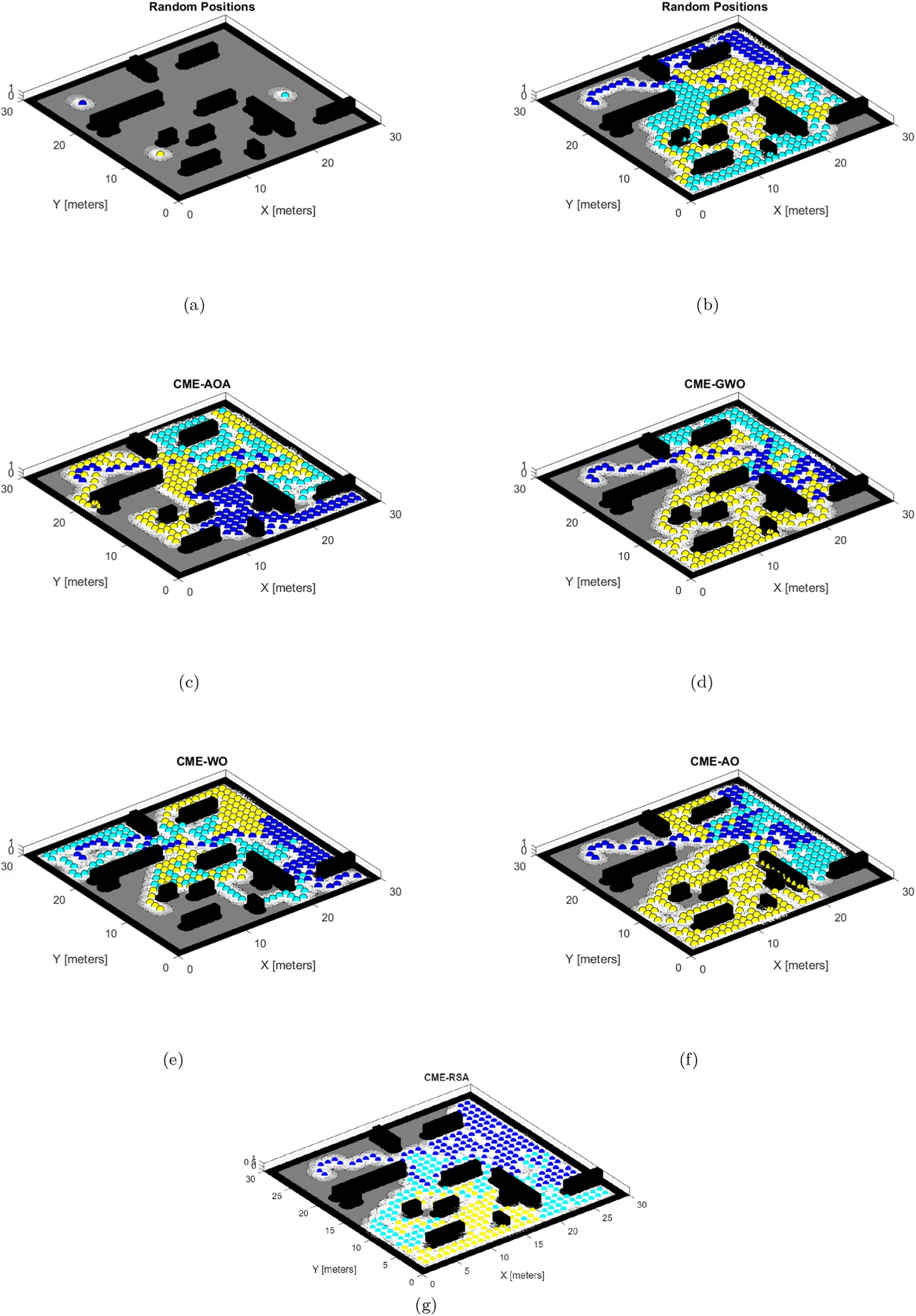
Random Positions of Obstacles in a Complex Map for EMREL and Contemporary Algorithm **(a)** The robots are randomly positioned in three different colors: blue, cyan, and yellow. Each color represents a robot in a homogeneous beginning setup, with distinct starting places and travel trajectories in the environment. **(a)**. **(b)** Random Positions EMREL 87.84%. **(c)** CME-AOA 81.95%. **(d)** CME-GWO 77.83%. **(e)** CME-WO 57.43%. **(f)** CME-AO 67.89%. **(g)** CME-RSA 79.34%.

**FIGURE 15:**
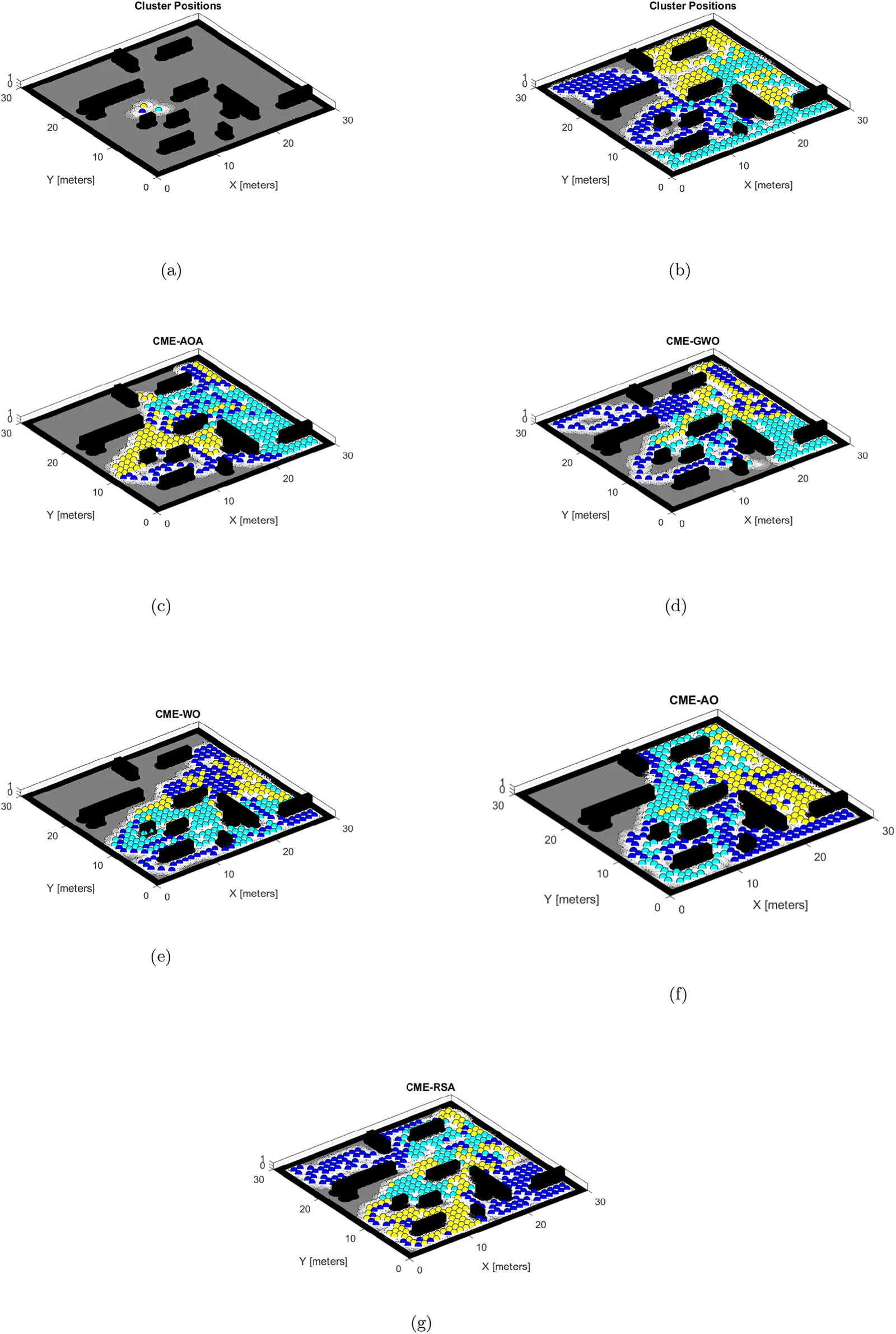
Cluster Positions of Obstacles in a Complex Map for EMREL and Contemporary Algorithm **(a)** Cluster positions of robots in three different colors: blue, cyan, and yellow. Each color represents a robot in a homogeneous beginning setup, with distinct starting places and travel trajectories in the environment. **(a)**. **(b)** Cluster Positions EMREL 93.93%. **(c)** CME-AOA 54.87%. **(d)** CME-GWO 78.87%. **(e)** CME-WO 45.98%. **(f)** CME-AO 71.71%. **(g)** CME-RSA 89.65%.

The first subfigure of all figures about complex instances depicts the robots’ initial positioning; refer to Figures 13a, 14a, 15a. This configuration serves as the foundation for all future tests. To ensure a fair comparison, each contemporary algorithm is tested with identical robot placements and map layout. Refer to Figures 13c, 14c, 15c for CME-AOA, Figures 13d, 14d, 15d for CME-GWO and CME-WO algorithm reviews Figures 13e, 14e, 15e. Similarly, for CME-AO algorithm refer to Figures 13f, 14f, 15f; for CME-RSA, refer to Figures 13g, 14g, 15g. This uniform testing methodology enables direct comparison of performance across many methodologies. Our proposed strategy regularly outperforms the others, displaying higher adaptability, efficiency, and exploration coverage in these difficult settings. The findings demonstrate the stability and effectiveness of our strategy, especially in complex contexts where strategic planning and coordinated exploration are crucial.

## 6 Final analysis, discussion, and results

This work utilizes a careful and diverse approach to evaluate several algorithmic solutions for multi-robot exploration systems. The data, presented in separate tables, focus on key performance measures such as exploration percentages, time efficiency, failure occurrences, and statistical significance. We use measurements such as mean (average) and standard deviation to comprehensively evaluate the algorithms’ effectiveness and dependability. A methodological design based on central limit theorem concepts supports the empirical foundation of this study. We tested each algorithm over 30 runs with 250 iterations each for a 30 × 30 m map and a 1.5 m sensor ray length to ensure the integrity and robustness of the findings. This design adheres to statistical best practices, aiming for a normal distribution that justifies the sample size and iteration count used for this study. We used the crayfish optimizer algorithm (COA), known for its inherent stochasticity, with heterogeneity among runs demonstrating methodological rigor. Understanding the computational complexity of multi-robot exploration algorithms is crucial for determining their efficiency. This statistic provides insight into the algorithms’ scalability and viability for real-world applications, particularly where speedy decision-making is critical. This systematically planned research method creates a large and varied dataset and gives the results a high level of reliability and accuracy, meeting the analytical needs of the highest level of scientific research. Because it has a good mix of rigorous methods, deep analysis, and sophisticated empirical work. The results we found show that starting configurations have a big effect on the exploration rate. However, we must examine these results in real-world scenarios where multiple factors simultaneously influence this rate. Table 2 caters to a summary of all experimental settings.

**TABLE 2:** An overview of the experimental cases used to evaluate EMREL.

Cases	Input configuration	Variation introduced	Evaluation focus	Expected outcome
Case 1 – Uniform Distribution	A 30 m × 30 m static map with fixed obstacles and evenly spaced robots	None (baseline configuration)	Performance of baseline exploration in optimal, uniform settings	Smooth trajectories, high coverage rate, and effective robot cooperation
Case 2 – Dynamic Obstacles	Environment with moving obstacles	Obstacle positions change dynamically during exploration	EMREL’s flexibility and real-time re-planning capabilities	Collision avoidance under dynamic changes, rapid path reconfiguration, and a steady exploration rate
Case 3 – Different Starting Positions	Three initial configurations: strategic, random, and clustered robot placements	Robot starting positions vary from trial to trial	Intelligent Initial Configuration’s (IIC) effect on the effectiveness of exploration	Performance that is consistent across all configurations, with decreased path overlap and faster convergence
Case 4 – Complex Map Layouts	Irregular environment with narrow passages and multiple obstacle clusters	Restricted spaces and intricate map topologies	Scalability, resilience, and collaborative effectiveness in complex settings	Better exploration coverage in intricate layouts, adaptive navigation, and efficient frontier updating

### 6.1 Exploration percentage

The application of average and standard deviation is critical when evaluating the performance of multi-robot exploration algorithms. The average exploration percentage is a clear and short description of an algorithm’s normal performance across numerous trials, indicating how precisely it explores the environment on average. This statistic provides for a simple comparison of different algorithms, revealing which ones performs superior overall. However, the standard deviation is equally essential because it measures the constancy of the algorithm’s performance. While a high average exploration % is ideal, it is as important to evaluate how consistent that performance is across different scenarios. A reduced standard deviation suggests the algorithm frequently performs near its average, implying resilience and reliability. In contrast, a higher standard deviation may indicate that the algorithm’s performance is more variable, which could be problematic in real-world applications where predictability and stability are critical. Together, these measures provide a comprehensive view of each algorithm’s effectiveness and reliability, allowing you to choose the optimum technique for multi-robot exploration tasks.

Analysis by case:

- Case 1: (Table 3) With its highest mean and lowest standard deviation, EMREL displays superior performance in both average exploration and stability. On the other hand, CME-WO lags in its ability to explore and exhibits higher levels of volatility.
- Case 2: (Table 3) Assessing algorithm performance gets increasingly complex in dynamic scenarios due to the continually changing variables. Although all algorithms exhibit a decline in exploration % due to the growing complexity of the scenario, EMREL consistently surpasses the performance of the other algorithms. The algorithm’s adaptability to changing contexts demonstrates its resilience, while other algorithms face difficulties in maintaining consistent performance in the face of variety. The ever-changing character of the case adds complexity to the evaluation process, as varying conditions make it more difficult to appropriately assess exploration and stability.

**TABLE 3:** Region explored in various cases.

Case No	EMREL		CME-AOA		CME-GWO		CME-WO		CME-AO		CME-RSA	
	% E (avg)	std	% E (avg)	std	% E (avg)	std	% E (avg)	std	% E (avg)	std	% E (avg)	std
1	98.07	1.25	78.95	3.76	85.65	2.87	85.90	6.86	87	7.89	95.78	2.98
2	92.87	2.98	78.94	7.54	81.95	5.79	71.35	9.54	79.65	5.78	87.53	3.67

### 6.2 Temporal performance

The average time required shows each algorithm’s computational efficiency, or how quickly it completes its duties. A shorter average duration indicates superior speed and computing efficiency. Meanwhile, the standard deviation indicates the constancy of this efficiency over numerous runs. A lower standard deviation indicates more consistent performance, whereas a higher one reflects variation in execution times.

Analysis by case: Cases 1–2 (Table 4): In the investigation of cases 1 and 2, EMREL regularly outperforms in terms of time efficiency, demonstrating its applicability for applications that require quick replies. CME-WO and CME-AO, on the other hand, have lower temporal efficiency, especially in case 2, which means they are more sensitive to environmental complexity.

**TABLE 4:** Explored region per second (Temporal Performance).

Case No	EMREL		CME-AOA		CME-GWO		CME-WO		CME-AO		CME-RSA	
	% E (avg)	std	% E (avg)	std	% E (avg)	std	% E (avg)	std	% E (avg)	std	% E (avg)	std
1	19.56	0.45	27.37	0.79	24.45	0.59	30.72	0.95	34.65	1.45	26.67	0.84
2	24.83	0.36	31.84	0.67	29.54	0.85	36.84	0.72	45.87	2.67	33.60	1.32

### 6.3 Failure analysis

Table 5: Failure rates provide useful information about an algorithm’s resilience, demonstrating how well it handles obstacles and unforeseen hurdles. Higher failure rates may indicate that an algorithm requires additional refining to improve reliability and performance.

**TABLE 5:** Failure rates.

Case No	EMREL	CME-AOA	CME-GWO	CME-WO	CME-AO	CME-RSA
1	0	4	2	7	8	4
2	0	1	1	3	4	3

Cases 1 and 2: In situations 1 and 2, EMREL performs efficiently with no faults, demonstrating its outstanding reliability. CME-WO, CME-AOA, and CME-RSA on the other hand, have higher failure rates, emphasizing the importance of increased resilience when dealing with complicated or unexpected situations.

### 6.4 Explored percentages for positions

Analysis by Case:

- Case 3 (Table 6): The cluster position layout drove the performance, resulting in a precise average investigated area of 99.19%. Other layouts, such as random and strategic placements, also proved effective, with exploration percentages ranging from 93.72% to 98.54%.
- Case 4 (Table 6): Strategic positions lead with a highest exploration rate of 92.59%. The random and clustered positions show varying performance levels, ranging from 87.84% to 93.93%, reflecting their adaptability to different environmental conditions.

**TABLE 6:** Explored regions in position cases.

Case No	Strategic position		Random positions		Cluster positions	
	% E (avg)	std	% E (avg)	std	% E (avg)	std
3	98.54	3.54	93.72	0.98	99.19	4.87
4	92.59	2.90	87.84	0.99	93.93	5.89

The table illustrates the efficacy of diverse investigation tactics in various contexts. Every position possesses distinct strengths and weaknesses, rendering them appropriate for specific conditions while proving less effective in others. The data from this table can guide future research in enhancing exploration tactics to more effectively correspond with particular scenarios, hence optimizing performance according to environmental requirements.

### 6.5 *p*-value analysis

This paper introduces a novel approach named EMREL in the domain of independent multi-robot exploration, aimed at functioning efficiently in environments of diverse complexity. The research employs an interdisciplinary approach that combines qualitative findings with rigorous quantitative analysis, offering a thorough understanding of spatial exploration. The research utilizes two primary metrics—mean and standard deviation—to measure area coverage and to analyze the reliability of the proposed method relative to established methodologies (refer to Tables 7, 8).

**TABLE 7:** *p*-values derived from the rank-sum test for the exploration data.

Case No	EMREL	CME-AOA	CME-GWO	CME-WO	CME-AO	CME-RSA
1	N/a	1.45x $10^{-2}$	5.73x $10^{-5}$	3.67x $10^{-4}$	2.68x $10^{-5}$	1.34x $10^{-3}$
4	N/a	2.53x $10^{-6}$	10.43x $10^{-9}$	8.43x $10^{-8}$	4.89x $10^{-8}$	1.87x $10^{-2}$

**TABLE 8:** Scenario Number: *p*-values Dimensions and Design of Robots: The utilization of Turtlebot 3, due to its particular dimensions and design parameters, may not accurately reflect larger or differently configured robots. The observed behaviors and outcomes may differ if an alternative robot design is utilized, as indicated by the rank-sum test results for time.

Case No	EMREL	CME-AOA	CME-GWO	CME-WO	CME-AO	CME-RSA
1	N/a	5.89x $10^{-12}$	8.34x $10^{-9}$	4.62x $10^{-7}$	3.42x $10^{-10}$	2.90x $10^{-4}$
4	N/a	6.43x $10^{-10}$	5.76x $10^{-13}$	3.78x $10^{-9}$	4.65x $10^{-10}$	2.70x $10^{-6}$

This study utilizes an advanced statistical framework, employing the Wilcoxon rank-sum test to examine individual experimental runs and reveal underlying trends. The analysis is organized around two primary hypotheses: H0, which asserts that the EMREL approach may exhibit inferior performance regarding exploration rate and time efficiency, and H1, which claims that it surpasses current methodologies.

The findings presented in Tables 7, 8 furnish compelling evidence to reject the null hypothesis, with p-values repeatedly falling below the significance level of 0.05. The findings are further substantiated by a systematic pairing and comparison of the top-performing approaches across several test functions, hence reinforcing the robustness of the conclusions.

This paper’s consistent methodological execution validates its analytical rigor and reinforces the legitimacy of its insights. The work adeptly combines qualitative depth with quantitative precision, positioning itself as a significant addition to multi-robot exploration and providing a substantial advancement in both theory and practice.

Statistical Significance: *p*-values for Exploration (Table 7).

1. The significance of p-values is in their capacity to provide statistical evidence concerning the importance of exploratory data. Decreased p-values signify a more robust rejection of the null hypothesis, indicating more persuasive evidence against it.
2. The results across multiple scenarios demonstrate statistically significant differences across the algorithms. The remarkably low p-values for CME-AOA, CME-GWO, and CME-WO underscore their unique performance.

Statistical Significance: p-values for Time (Table 8).

1. Significance: The p-values related to time metrics provide statistical evidence for evaluating the efficiency of various algorithms, assessing the probability that observed differences occurred by chance.
2. Analysis: Statistically substantial variations in exploration data and temporal efficiency may indicate inherent computational complexity or design discrepancies. Algorithms exhibiting consistent superiority are likely more efficient in managing computational requirements. Observations: The remarkably low p-values noted in all comparisons highlight significant differences in time efficiency across the algorithms, confirming that their performance variances are not coincidental but stem from structural or operational differences.

#### 6.5.1 Balancing exploration speed and coverage

1. Hierarchical Planning Framework:
2. Global Coverage Paths: The program initially determines effective global coverage paths by utilizing the deterministic method, which retains essential environmental data. This guarantees that the robots favor uncharted regions, optimizing coverage.
3. Local Refinement: After establishing a global path, the algorithm enhances the investigation by identifying ideal local perspectives. This stage guarantees that the robots meticulously investigate areas of interest, improving coverage without considerably impeding the exploration process.
4. Minimum-Time Trajectories: The final phase entails the generation of safe, dynamically feasible, and minimum-time paths. This guarantees that the robots navigate swiftly while circumventing impediments, enhancing exploration efficiency.
5. Stochastic Optimization: The Crayfish Optimization Algorithm (COA) is employed to enhance the exploration process. By probabilistically choosing the optimal subsequent views and trajectories, the algorithm balances the necessity for swift exploration with the demand for extensive coverage. Utility function within the frontier structure guarantees that robots prioritize unexplored regions with significant information gain. As robots navigate these locations, the utility values diminish, directing them to new regions and ensuring extensive coverage.
6. Dynamic Adaptation: The algorithm adjusts dynamically to the complexity of the environment. In uncluttered surroundings, robots may navigate swiftly with minimal adjustment. In intricate situations, the algorithm enhances the degree of local refinement to guarantee comprehensive coverage.

#### 6.5.2 Trade-off management

1. Exploration Rate vs. Coverage: The method employs a cost-utility trade-off to optimize the balance between exploration velocity and coverage. By reducing movement costs and enhancing the utility of uncharted regions, the algorithm guarantees that robots explore well without compromising coverage.
2. Simulation Results: Our simulations indicate that EMREL attains elevated coverage rates (e.g., 98.54% in strategic positions) while executing exploration in a reduced time frame relative to competing algorithms. This signifies that the algorithm adeptly reconciles speed and coverage.
3. Complex Environments: In intricate situations, the algorithm enhances local refinement to guarantee comprehensive exploration of complex regions. This may marginally decrease exploration velocity but guarantees extensive coverage.
4. Dynamic Environment: The algorithm’s capacity to adjust to dynamic impediments significantly improves its efficacy in intricate situations, ensuring extensive coverage while maximizing exploration efficiency.
5. Algorithm Efficiency: The EMREL algorithm proficiently equilibrates exploration velocity and coverage via its hierarchical planning framework, stochastic optimization, and dynamic adaptation features. By emphasizing unexplored regions and creating efficient paths, the algorithm guarantees extensive coverage while maximizing centralized exploration.

## 7 Integration of proposed method with ROS2 for autonomous navigation

The article effectively combines the suggested EMREL framework with ROS2, showcasing its versatility in TurtleBot3 (TB3) simulations. The implementation comprises two ROS2 nodes for autonomous navigation, facilitating accurate trajectory control in Gazebo. The modular node-based architecture improves scalability and maintainability, while ROS2’s sophisticated connectivity guarantees dependable real-time operations. Testing on both standard and custom TB3 maps shows accurate navigation with only small errors, showing that the method works in various settings. The amalgamation of MATLAB and RViz with Gazebo enables centralized management and real-time visualization of multi-robot exploration. Using the gmapping and AMCL packages ensures accurate mapping and localization. The system demonstrates potential for intricate exploratory tasks, with forthcoming efforts focused on enhancing coordination algorithms and broadening testing situations.

### 7.1 Combining MATLAB and RViz to integrate several TurtleBot3 robots for joint research in a gazebo setting

Gazebo and MATLAB are combined in the integration process for multi-robot exploration. To ensure spatial compatibility, a 30 × 30 m MATLAB map is aligned with Gazebo using linear transformations. While exploring their surroundings, autonomous TurtleBot3 robots send positional data to MATLAB for centralized coordinating. The robots in Gazebo receive the synchronized movement orders that MATLAB generates from this data (Figures 16a–c). Until the exploration is finished, this cycle is repeated. Real-time robot placement and precise environment reconstruction are made possible by the gmapping and AMCL packages, which accomplish mapping and localization (Figures 16d–f). Autonomous navigation is made possible by the movebase package, which enables effective traversal, dynamic path planning, and obstacle avoidance. Strong spatial awareness and fluid navigation in challenging surroundings are demonstrated by this integration.

**FIGURE 16:**
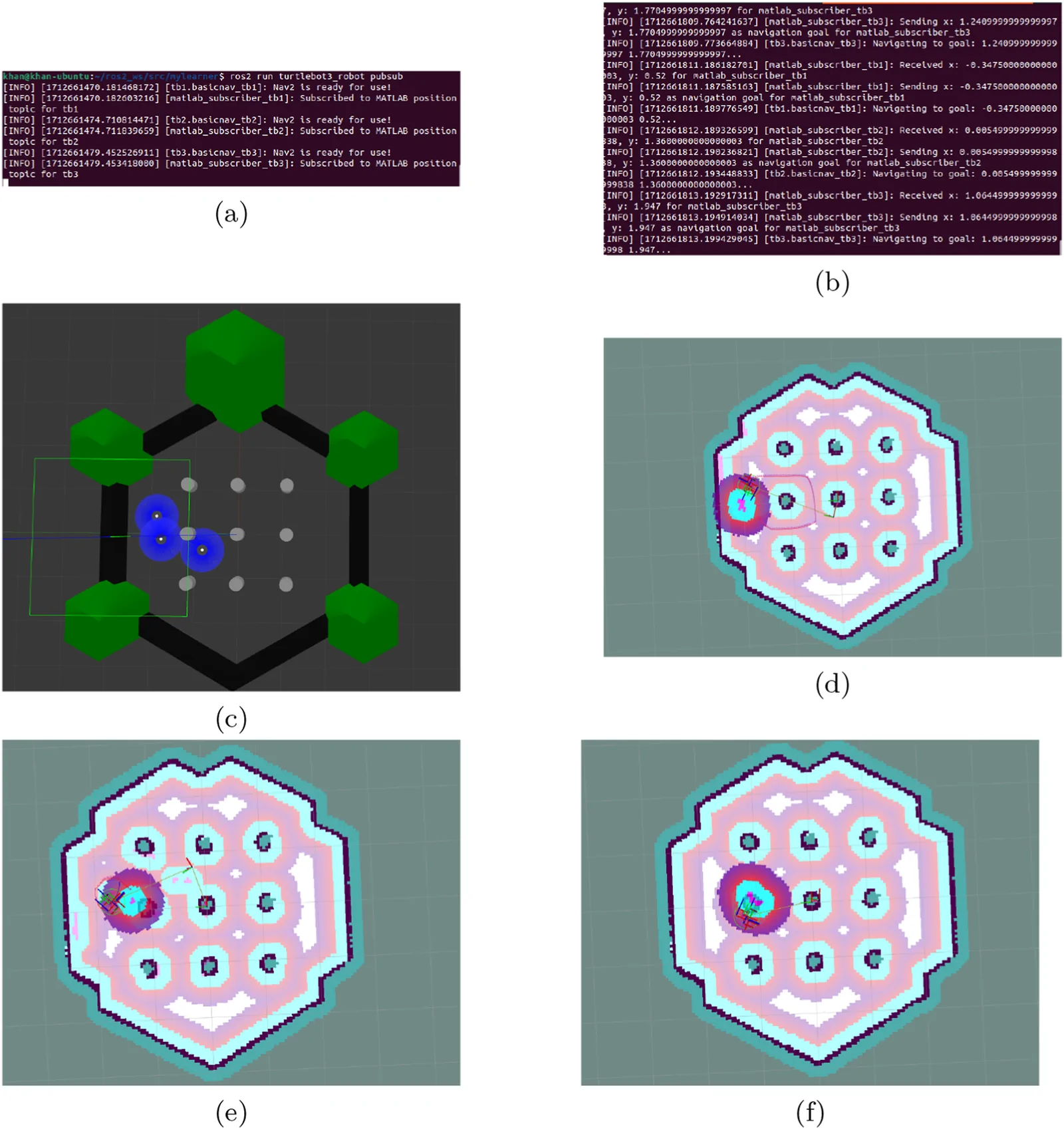
Gazebo Simulation Environment Setting 1 for Evaluating Performance, **(a)** Running a Publisher-Subscriber node **(b)** Node running, **(c)** Three turtlebots’ placement in Gazebo, **(d-f)** Rviz2 for each individual robot.

Figures 17a–d and Figures 18a–d show a Gazebo environment that is used to test the suggested framework. Three TurtleBot3 robots navigate the map on their own, simulating navigation in the real world. Real-time display of the robots’ trajectories, sensor outputs, and navigation metrics is made possible by their individual Rviz2 configurations. This configuration offers a regulated, repeatable platform to evaluate the robots’ cooperation, obstacle avoidance, and exploration effectiveness. Their behavior can be examined to improve system robustness and navigation algorithm performance. The outcomes demonstrate how well the framework can handle challenging multi-robot exploration tasks in changing conditions. Enhancing inter-robot cooperation, refining exploration tactics, and verifying the system through prolonged real-world testing could be the main goals of future research. All things considered, the combination of the TurtleBot3 robots with MATLAB, Gazebo, and Rviz2 creates a scalable basis for developing autonomous multi-robot systems in many contexts.

**FIGURE 17:**
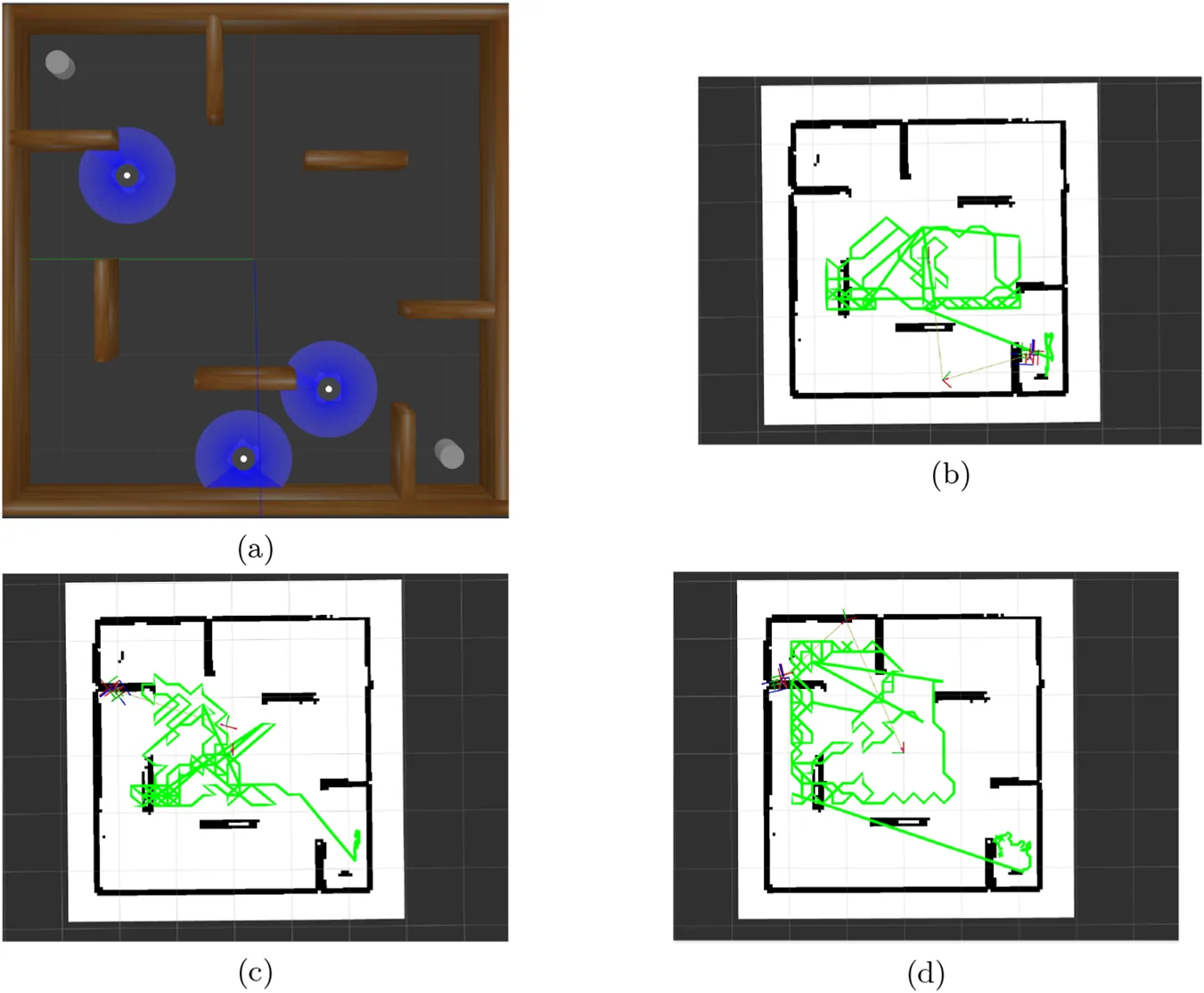
Gazebo Simulation Environment Setting 2 for Evaluating Performance, **(a)** Three turtlebots’ placement in Gazebo, **(b-d)** Rviz2 for each individual robot.

**FIGURE 18:**
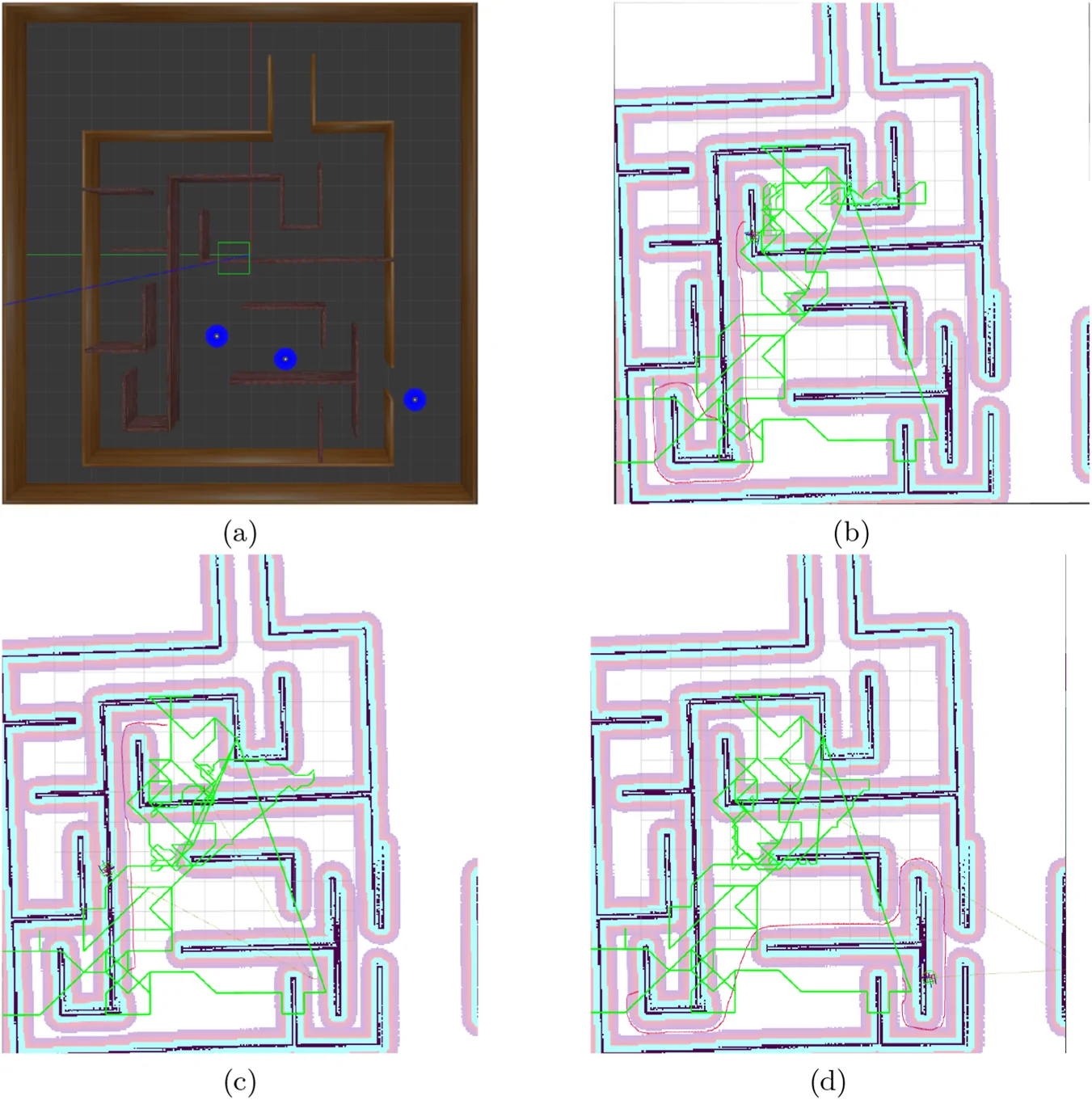
Gazebo Simulation Environment Setting 3 for Evaluating Performance, **(a)** Three turtlebots’ placement in Gazebo, **(b-d)** Rviz2 for each individual robot.

### 7.2 Gazebo results discussion

Using three TurtleBot3 robots along with MATLAB-based coordination, RViz visualization, and the Gazebo simulation environment led to positive outcomes in tasks requiring cooperative exploration. The integration of MATLAB with gazebo worked seemlessly. Every TurtleBot3 robot independently investigated its surroundings while sending positional information to MATLAB for centralized management. The robots could jointly investigate the area due to the synchronized movement commands supplied by the coordination algorithms in MATLAB. Additionally, the interaction with RViz made it possible to visualize the exploration process in real-time, enabling researchers to monitor and assess the effectiveness of the exploration tactics. Refer to Table 9 for performance comparison. The percentage of map coverage attained in MATLAB exceeds that of Gazebo, attributable to the duration required for the transmission of commands from MATLAB to Gazebo, as well as the execution time needed to accomplish a designated objective, such as reaching a specified target. Successfully completing 100 iterations without aborting the objective constitutes a noteworthy achievement, underscoring the resilience and efficacy of the implemented system.

**TABLE 9:** Comparison of algorithm for space exploration by autonomous robot.

Environment	Explored area in MATLAB	Elapsed time (minutes)	Explored area in Gazebo
Environment 1	93%	11	87.13%
Environment 2	96%	9	84.05%
Environment 3	92%	15	77.34%

#### 7.2.1 Real-world real-world hardware considerations

The integration of the EMREL algorithm with ROS2 and Gazebo showcases its efficacy in simulations; nevertheless, real-world hardware implementations may present obstacles like communication delays, computing constraints, and sensor errors. These characteristics may influence the algorithm’s efficacy, especially in dynamic settings with mobile barriers. To resolve these challenges, feasible methods encompass optimizing communication protocols, diminishing computational burden via algorithm simplification, and integrating robust sensor fusion approaches. Moreover, energy-conscious planning and comprehensive testing on physical robots are crucial to guarantee dependable performance in practical situations. Subsequent research should concentrate on verifying the algorithm’s scalability and flexibility in real-world applications.

#### 7.2.2 Limitations

The paper introduces the EMREL algorithm designed for effective multi-robot exploration in intricate situations. The algorithm exhibits notable enhancements in exploration efficiency, coverage, and adaptability; nonetheless, it possesses certain constraints. The primary limits of the algorithm, as articulated in the study, are as follows:

- Scalability to Larger Robot Teams: The technique was evaluated using a restricted quantity of robots, specifically three TurtleBot3 robots in Gazebo simulations. Although the results are encouraging, the algorithm’s scalability to bigger robotic teams remains unvalidated. As the quantity of robots escalates, the overhead associated with coordination and communication may intensify, thus diminishing the algorithm’s efficiency in extensive deployments.
- Real-World Environmental Complexity: The method was assessed in simulated settings featuring dynamic impediments and intricate configurations. Nonetheless, real-world surroundings frequently pose further hurdles, like irregular topography, fluctuating lighting conditions, and unforeseen dynamic barriers. The algorithm’s efficacy in real-world contexts has not been thoroughly evaluated, hence constraining its generalizability.
- Computational Complexity: The approach depends on stochastic optimization and hierarchical planning, which may be computationally demanding. While the technique exhibits excellent temporal performance in simulations, its computational requirements may escalate considerably in larger or more intricate situations. This may present difficulties for real-time applications with constrained processing resources.
- Communication Constraints: The algorithm presupposes dependable communication among robots for coordination and information exchange. In practical situations, communication may be compromised by interference, restricted range, or obstructions. The algorithm’s efficacy under these communication restrictions has not been extensively examined, potentially affecting its practical applications.
- Adaptability to Different Robot Platforms: The method was predominantly evaluated using TurtleBot3 robots. Its efficacy may fluctuate when used on diverse robotic platforms with differing dimensions, sensor proficiencies, and mobility limitations. The algorithm’s adaptation to various robotic systems is yet uncertain, potentially impacting its wider applicability.
- Extensive three-D Envirnoment: Three-dimensional environments present extra complications for path planning and obstacle avoidance, necessitating further examination.

## 8 Conclusion

In this research, we introduce a hybrid crayfish exploration technique with intelligent initial configuration. We have demonstrated a dynamic approach for optimizing exploration performance across various conceivable setups such as uniform distribution, dynamic movements, clustered positions, random positions, and strategic positions. The proposed hybrid coordinated architecture for multi-robot exploration for unexplored areas is aided by CME (deterministic method) and COA (stochastic optimization method). An integral element of our strategy is CME, which progressively updates the frontier points, supplying critical data to guide exploration planning. The stochastic method explores the area by tuning parameters. Our methodology facilitates rapid decision-making, enabling the multi-robot to swiftly adapt to environmental alterations. Both simulation benchmarks and empirical evaluations validate the efficacy of our methodology. We assessed key performance indicators such as runtime, explored area, and failure rates, highlighting EMREL’s considerable validity and efficiency. In rescue operations where human agents cannot modify the obstacle locations or primary robot positions, the proposed method can mitigate the challenges and shortcomings of traditional exploration methods.

## Data Availability

The raw data supporting the conclusions of this article will be made available by the authors, without undue reservation.
